# Adaptation of a methanogen to Fe^0^ corrosion via direct contact

**DOI:** 10.1038/s41522-024-00574-w

**Published:** 2024-10-04

**Authors:** Satoshi Kawaichi, Rhitu Kotoky, Jacek Fiutowski, Amelia-Elena Rotaru

**Affiliations:** 1 Department of Biology, University of Southern Denmark, Odense, Denmark; 2 NanoSYD, Mads Clausen Institute, University of Southern Denmark, Sønderborg, Denmark

**Keywords:** Environmental microbiology, Applied microbiology

## Abstract

Due to unique genomic adaptations, *Methanococcus maripaludis* Mic1c10 is highly corrosive when in direct contact with Fe^0^. A critical adaptation involves increased glycosylation of an extracellular [NiFe]-hydrogenase, facilitating its anchoring to cell surface proteins. Corrosive strains adapt to the constructed environment via horizontal gene transfer while retaining ancestral genes important for intraspecies competition and surface attachment. This calls for a reevaluation of how the built environment impacts methane cycling.

Iron (Fe^0^) corrosion in anoxic environments can be caused by methanogenic archaea, working alone or in conjunction with bacteria. Certain strains of *Methanococcus maripaludis* are particularly corrosive and are found to be associated with infrastructure corrosion globally^1^.

Previous studies on mutant strains of a non-corrosive *M. maripaludis* S2 indicated that biological corrosion occurs due to the release of free enzymes from moribund cells during the late-stationary phase^2^. These enzymes, such as extracellular hydrogenases, can catalyze electron uptake from Fe^0^ and hydrogen formation^3^. The hydrogen is then used as an electron donor by active cells of *M. maripaludis*^2^.

Further studies have suggested that free enzymes released by a severely corrosive *M. maripaludis* OS7 are the primary cause for its corrosive capabilities^4^. The strain contains a conserved yet genetically unstable genetic island, the Microbiologically Influenced Corrosion Island (MIC Island), with genes for two subunits of a [NiFe]-hydrogenase and the Tat transmembrane protein secretion system, believed to export the hydrogenases outside the cell. Researchers found [NiFe]-hydrogenases in spent cell filtrates and determined that the spontaneous deletion of the MIC-island in a mutant strain OS7mut1 resulted in a loss of the ability to corrode. This highlights the crucial role this island plays in corrosion^4^.

Moreover, when a segment of the MIC-island that included the [NiFe]-hydrogenases and the Tat-system was transplanted from the corrosive strain OS7 to a non-corrosive strain JJ, the resulting mutant exhibited some corrosive properties^5^, albeit an order of magnitude lower than corrosive OS7. This suggests there might be additional factors required for full corrosive potency. Complementing this insight, Holten et al.^5^ showed that glycosylated S-layer proteins facilitate the attachment of *M. maripaludis* to surfaces, including Fe^0^.

While previous studies indicate that corrosive *M. maripaludis* strains rely on free enzymes to access Fe^0^-electrons, free enzymes are costly as the producer makes them readily available to the entire community, including ‘cheater’ populations^6^. The cost is particularly high when such enzymes produce a highly diffusible product like hydrogen, which becomes a “public good”^6^. Therefore, retaining the enzymes close to the producer cells can provide a competitive advantage against ‘cheater’ populations.

Thus, we hypothesize that highly corrosive strains of *M. maripaludis* keep their extracellular enzymes on the cell surface, necessitating direct contact to the Fe^0^-surface. This enables effective electron uptake from Fe^0^ and corrosion. To test this hypothesis, we conducted physiological experiments with a highly corrosive strain, Mic1c10, originating from the walls of an oil storage tank^7^, sequenced its genome and compared it with three corrosive^1,4,8^ and three non-corrosive^9,10^ *M. maripaludis* strains. Our results show that corrosive cells interact directly with the metal surface to retrieve electrons, likely through [NiFe]-hydrogenases attached to cell surface proteins via glycan-glycan interactions.

## Mic1c10 Required Direct Cell-Fe^0^ Contact, Not Free Enzymes

**Fig. 1:**
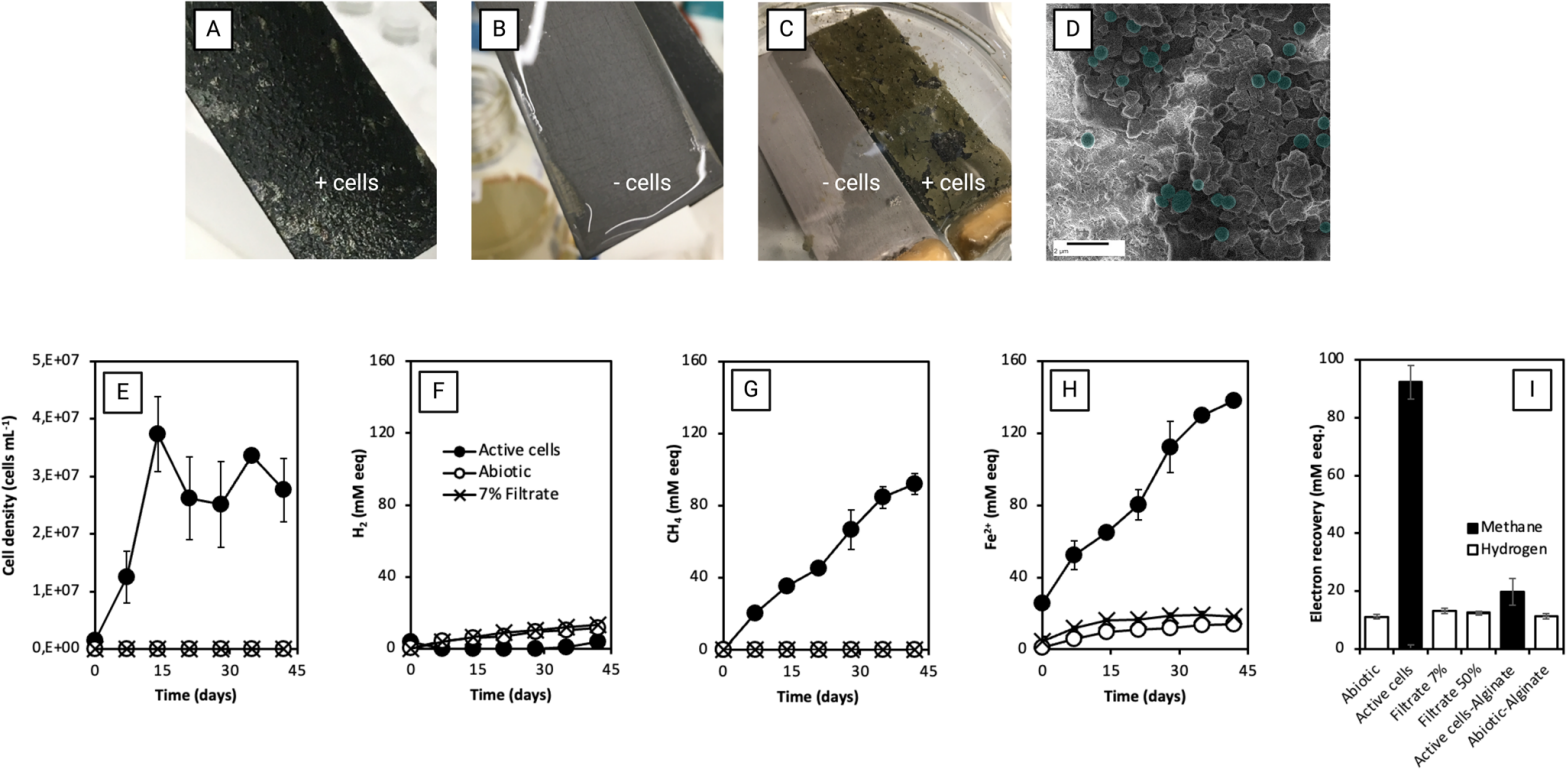
Corrosion by *Methanococcus maripaludis* Mic1c10. (**A**) Carbon steel corrosion by active cells versus (**B**) heat-killed cells, before (**A**, **B**) and (**C**) after acid-washing to remove loosely adhered, material. (**D**) helium ion microscopy image showcasing Mic1c10 colonization on Fe^0^-foil. (**E**) Cell quantification using DAPI staining after vigorous shacking to detach cells from Fe^0^-foil. (**F**–**H**) Measurement of corrosion products: hydrogen, methane, and ferrous iron (in mM electron equivalents). The mM eeq are calculated by multiplying the mM H_2_ by 2 (2e^−^ + 2H^+^ → H_2_), the mM methane by 8 (8e^−^ + 8H^+^ + CO_2_  → CH_4_ + 2H_2_O) and the mM Fe^2+^ by 2 (Fe^0^ → Fe^2+^ + 2e^−^) (**I**) Electron recovery comparison across treatments (see Supplementary Table 2). All tests were performed with several biological replicates (n ≥ 3).

We found that physical contact was crucial for corrosion by *M. maripaludis* strain Mic1c10. When Mic1c10 interacted with a carbon steel surface, it created a distinctive black film and caused permanent surface alterations to the material surface, which were not observed with heat-killed controls (Fig. 1A–C). With its small cell size ( ~ 500 nm diameter) and high surface-to-volume ratio, it could effectively attach, colonize, and corrode the metal surface (Fig. 1D, Supplementary Table 1). The number of Mic1c10 cells doubled every 2.5 days until they reached a plateau after two weeks. The cell count remained steady for another four weeks (Fig. 1E).

Methane and Fe^2+^ were the primary products of Mic1c10’s corrosive metabolism over six weeks (Fig. 1F–H). This was due to the methanogenic metabolism of Mic1c10, which drove the reduction of CO_2_ to methane and ferrous iron (Fe^2+^) via the MIC reaction (4Fe^0^ + 5HCO_3_^−^ + 5H^+^ → CH_4_ + 4FeCO_3_ + 3H_2_O). On the contrary, abiotic controls accumulated hydrogen and Fe^2+^ according to an abiotic reaction (Fe^0^ + HCO_3_^−^ + H^+^ → FeCO_3_ + H_2_). The cathodic depolarization theory suggests that microorganisms enhance corrosion by consuming this H_2_, reducing its concentration at the Fe^0^ surface to make corrosion more thermodynamically favorable^11^. However, we found that a non-corrosive strain JJ maintained similar low hydrogen levels as the corrosive Mic1c10 (Supplementary Fig. 1), suggesting that Mic1c10 may use an alternative strategy - extracellular enzymes - for effective electron uptake from Fe^0^.

We then analyzed the spent filtrate, which is expected to contain extracellular enzymes that promote Fe^0^ corrosion even without active cells^2,4^. However, the addition of spent filtrate from Mic1c10 to fresh media with Fe^0^ resulted in negligible H_2_- and formate-buildup (0.16 ± 0.04 mM), which could explain less than 3% of the methane formed by cells (Fig. 1). This was independent of whether the filtrate was from a 1-month-old (stationary) or 2-weeks-old (exponential) culture (Supplementary Fig. 2). Cyclic voltammetry confirmed that spent filtrate was ineffective at promoting H_2_ formation (Supplementary Fig. 3). Furthermore, spent filtrate did not promote accumulation of Fe^2+^, a direct product of Fe^0^-oxidation, above abiotic controls (*p* = 0.45, *n* > 3), clearly showing that Mic1c10 filtrate lacked intrinsic corrosive properties. These findings are in contrast to earlier reports^2,4^ that highlighted the corrosive effect of free enzymes. We thus directed our focus to cell-bound elements as potential primary agents for severe corrosion, since soluble extracellular constituents in Mic1c10 spent filtrate had minimal impact.

To investigate whether physical contact is necessary for cells to induce Fe^0^ corrosion, we applied an alginate hydrogel coating on the Fe^0^ surface, which allows molecular diffusion of hydrogen while blocking cell contact with the metal. We observed that cells interacting with alginate-coated-Fe^0^ exhibited a fivefold decrease in methane production compared to those in direct contact with uncoated Fe^0^ (Fig. 1I, Supplementary Fig. 4). This suggests that Mic1c10 was inefficient in extracting electrons from Fe^0^ without direct contact, and together with the filtrate experiments emphasize the critical role of physical interaction in the microbial corrosion mechanism.

The next objective was to understand what makes Mic1c10 cells efficiently extract electrons from Fe^0^, leading to severe corrosion. We hypothesized that Mic1c10 uses surface-anchored hydrogenases to facilitate electron uptake from Fe^0^. To test this hypothesis and elucidate the genetic basis of its severely corrosive behavior, we conducted a comparative genomic analysis. We sequenced Mic1c10’s genome (Supplementary Table 3) and compared it to non-corrosive (JJ^9,10^, C5 and S2^10^) and corrosive *M. maripaludis* strains (KA1^8^ and OS7^4^) as well as a nearly complete metagenome-assembled genome (MAG – MIC098Bin5) retrieved from corroded infrastructure^1^.

## Core genome of corrosive strains and adaptive traits

**Fig. 2:**
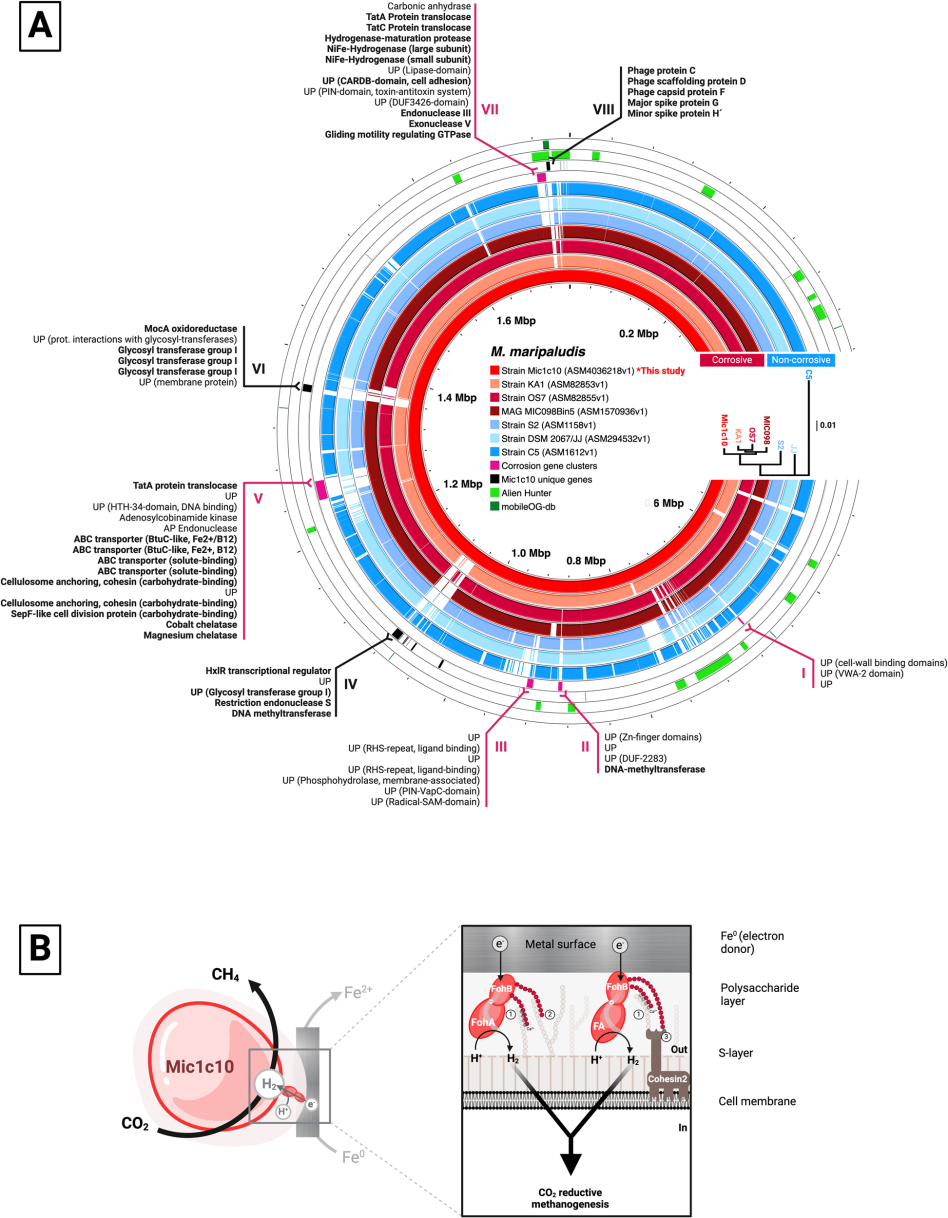
Comparative genomics of the newly sequenced *Methanococcus maripaludis* Mic1c10 against corrosive and non-corrosive strains. **A** Concatenated maximum likelihood phylogeny of 447 core genes (insert) and whole genome alignments highlighting divergent genetic regions. Red inner rings represent corrosive strains, while blue rings indicate non-corrosive strains. Pink highlights gene islands shared between all corrosive strains. Black indicates gene islands unique to Mic1c10. Light green shows regions likely acquired through horizontal gene transfer (see all HGT-maps in Supplementary Fig. 7), and dark green marks mobile genetic elements. UP stands for uncharacterized proteins. **B** Predicted model of the two subunits of Fe^0^-oxidizing hydrogenases (FohAB) anchoring on the cell surface. (1) Ca^2+^-dependent glycan-glycan interactions, (2) polyvalent zipper glycan-glycan interactions, (3) carbohydrate-binding-domain on protein–glycan interaction.

Our analysis revealed a close evolutionary relationship among corrosive strains based on 447 core genes (Supplementary Fig. 5), while non-corrosive strains showed a marked divergence (Fig. 2A-insert). Average Nucleotide Identity (ANI) values corroborated these findings with Mic1c10 sharing high ANI ( ≥ 98.5%) with other corrosive strains and lower ANI ( < 97%) with non-corrosive strains (Supplementary Fig. 6). Mic1c10’s similarity to the environmental MAG (MIC098Bin5), which has been globally linked to infrastructure corrosion, indicates a shared evolutionary adaptation to the built environment and underscores the ecological relevance of our findings.

Next, by using comparative genomics we identified several conserved syntenic genomic regions (islands) specific to corrosive strains (island I, II, III, V and VII) and some unique to Mic1c10 (islands IV, VI, VIII) (Fig. 2, Supplementary Fig. 7, Supplementary Tables 4-5). Most of these islands encode for functions that appear critical for the corrosive capabilities of the strains, suggesting that the ability to cause severe corrosion is a genetically determined trait.

Island I encodes extracellular proteins, including an uncharacterized cell wall-binding protein and a VWA2 (Von Willebrand Factor)-domain protein. These VWA2-proteins are involved in cell adhesion in eukaryotes^12^ and regulate archaella expression in archaea^13^. Island III contains genes coding for extracellular or cell membrane-proteins, including two RHS (rearrangement hot spot) proteins^14^ and a PINc-domain/VapC-family (virulence associated protein C)-protein^15,16^, typical of polymorphic toxin-antitoxin systems used for inter-strain competition^17^. Island V encodes for type II cohesins (cellulosome-anchoring) and transporters for siderophores, solutes, vitamins and cations like Mg^2+^. In bacteria, cellulosome complexes utilize type II cohesins to attach to the cell surface^18^. Similar cohesins have been identified in the non-cellulosomal *Archaeoglobus fulgidus* though their function remains unclear^19^. We suggest that in Mic1c10 and other corrosive strains, these cohesins may facilitate the anchoring of extracellular enzymes, such as [NiFe]-hydrogenases.

Island VII, or the MIC island, encodes the two subunits of a secreted bidirectional [NiFe]-hydrogenase^4^, which we refer to here as Fe^0^-oxidizing hydrogenases (FohAB). Our *in-silico* predictions suggest that these subunits form a dimer with external protrusions that could be glycosylated. These subunits have nine N-x-T/S motifs specifying glycosylation sites (Supplementary Table 4). The [NiFe]-hydrogenase of Mic1c10 has four additional glycosylation sites compared to other corrosive strains (Supplementary Fig. 8), suggesting it may become more adhesive when fully glycosylated. This tight anchorage could explain why hydrogenase activity was deficient in Mic1c10 cell filtrates, but not in OS7’s cell filtrates^4^.

Glycosylated FohAB-hydrogenases may attach to the cell surface through two primary mechanisms: protein-glycan interactions involving carbohydrate-binding proteins encoded on island V, or glycan-glycan interactions with glycosylated type II cohesins or S-layer proteins (Fig. 2B). These glycan-glycan interactions could either be direct polyvalent interactions or facilitated by Ca^2+^-ionic bridges^20,21^. Corrosive strains exhibit the highest density of glycosylation sites on their type II cohesins and S-layer proteins (Supplementary Tables 4 and 6).

In *M. maripaludis*, glycosylation of cell surface proteins typically involves glycosyltransferases like AglB (archaeal glycosyltransferase), which is not unique to corrosive strains. Additionally, Mic1c10 has a unique gene cluster (island VI) rich in glycosyltransferase genes (Supplementary Tables 5 and 7). The combination of additional glycosyltransferases, and high glycosylation potential of hydrogenases and cell surface proteins, may explain Mic1c10’s lack of hydrogenase activity in cell supernatants^22,23^ (Fig. 1I). These results support the hypothesis that Mic1c10 utilizes surface-anchored hydrogenases to facilitate electron uptake from Fe^0^ (Fig. 2). The observed intraspecies diversity of *M. maripaludis* indicates a significant adaptive potential in Mic1c10, where minor genetic variations, such as additional glycosylation sites on its hydrogenase, can influence its phenotypic behavior.

## Emergence of corrosion-specific gene clusters

Next, we investigated the role of mobile genetic elements and horizontal gene transfer (HGT) events in the emergence of corrosion specific gene clusters (Fig. 2A, Supplementary Fig. 9). Our analyses suggest that the MIC island VII (specific to corrosive strains), and island VIII (unique to Mic1c10) were acquired through HGT. This process facilitates the rapid acquisition of new genes including FohAB-hydrogenases, which enables corrosive strains to adaptively evolve and exploit a new ecological niche.

The origin of the other six islands was less straightforward. Many genes showed clear archaeal ancestry or traced back to a common ancestor, likely emerging through vertical inheritance and subsequent diversifications. Non-corrosive strains likely lost these islands due to weak selection pressure in their nutrient-rich, stable salt marsh environments^24^. In such environments, genes that do not contribute to survival are often lost because keeping unnecessary genes is energetically costly^25^. Conversely, corrosive strains may have retained some ancestral gene clusters, and thus maintained core adaptations.

Thus, corroders have likely adapted to the built environment through rapid environmental adaptation following one or two HGT events, while also retaining ancestral genes that could be crucial in intraspecies competition, and extracellular attachment.

## Conclusion

Mic1c10 required direct contact to Fe^0^ to uptake electrons, and showed insignificant enzymatic activity by cell filtrate, thus challenging the view that free enzymes primarily drive corrosion by *Methanococcus*. We identified corrosion-specific gene islands, containing genes for [NiFe]-hydrogenases, cohesins, transporters and toxin-antitoxin systems crucial for inter-strain competition. Mic1c10’s unique glycosyltransferases, and highly glycosylatable [NiFe]-hydrogenases (FohAB) and cell surface proteins, could explain how hydrogenases remain anchored to the cell surface and do not act as free enzymes in the cell filtrate. The acquisition of corrosive traits via horizontal gene transfer (the MIC island), coupled with the retention of ancestral genes in all known *M. maripaludis* corrosive strains, highlights their adaptability to the constructed environment. These findings add to our understanding of microbially-influenced corrosion and may inform strategies for its control.

## Methods

### Strains and cultivation conditions

*M. maripaludis* Mic1c10 ( = NBRC 105639)^7^ was routinely cultured on NBRC medium 927 with the following modifications: reduced amount of Fe^0^ granules to 100 g/L, and addition of 1 mg/L resazurin as an oxygen indicator. The medium and stock solutions were prepared anaerobically by degassing with N_2_/CO_2_ (80/20). All culture experiments were carried out in 120 mL serum bottles containing 50 mL culture, a gas phase of N_2_/CO_2_ (80/20), and 5.0 g Fe^0^ granules (1 to 2 mm in diameter, 99.98% purity metal basis; Alpha Aesar, Ward Hill, MA) at 37 °C unless otherwise mentioned. Any culture showing signs of oxygen contamination was removed from the experiments. Amount of CH_4_, H_2_, and Fe^2+^, and the cell density were measured periodically (see below).

To assess possible artifacts regarding the impact of inactivated cellular material on corrosion (e.g., during electrochemical corrosion measurements and electron microscopy), we performed additional incubations using heat-killed cells. In these experiments, mid-exponential phase Mic1c10 cells were heat-killed by autoclaving at 121°C for 30 minutes. The heat-inactivated cells were then used as inoculate for the heat-killed control incubations.

### Preparation of cell-free spent culture medium (SCM)

SCM was prepared by filtering Fe^0^-grown one-month-old (early stationary phase) and 2-week-old cultures (exponential phase) through two stacked syringe filters (cellulose acetate membrane, 0.2 μm pore size; GVS Filter Technology), essentially as described by Deutzmann et al.^2^. To initiate the experiment, 3 and 25 mL of SCM was added anoxically to 40- and 25-mL fresh medium, respectively, with 5.0 g Fe^0^ granules.

### Encapsulation of Fe^0^ with alginate hydrogel

For the alginate experiment, 5 mL of 1% (w/v) alginic acid sodium salt solution was added to a 120 mL serum bottle with 5.0 g iron granules. Then the bottle was degassed with N_2_/CO_2_ (80/20) for 10 minutes and autoclaved. Ten milliliters of 1% (w/v) CaCl_2_ solution was slowly added to the bottle to overlay on the alginic acid solution, and the bottle was allowed to stand still overnight to solidify the hydrogel. The remaining CaCl_2_ solution was removed, and the solidified hydrogel was washed twice with 10 mL of fresh medium. Finally, 45 mL of fresh medium was added to the bottle. To initiate the experiment, 5 mL of well-grown culture was added to the bottle.

### Analytical procedures

CH_4_ and H_2_ were measured using a gas chromatograph (GC) (Trace 1300, Thermo Scientific) equipped with a thermal conductivity detector (TCD). The injector was operated at 150 °C and the TCD at 200 °C with 1.0 mL/min reference gas flow. The oven temperature was constant at 70 °C. Gas chromatography analyses used a TG-BOND Msieve 5 A column (30-m length, 0.53-mm i.d., and 20-μm film thickness, Thermo Scientific) with argon carrier gas at 25 mL/min. The GC was controlled and automated by the Chromeleon software (Dionex, Version 7).

The amount of Fe^2+^ was measured using a modified ferrozine method^26^. A 100 μL sample was acidified with 900 μL of 0.5 M HCl, 10 μL of the mixture was added to 2 mL of 0.2 g/L 3-(2-pyridil)-5,6-bis(4-phenylsulphonate)-1,2,4-triazin (ferrozine) solution in 50 mM HEPES at pH 7.0, and the absorbance at 562 nm was measured.

Cell growth was monitored by the direct cell count of 4’,6-diamidino-2-phenylindole (DAPI) stained cells using an epifluorescent microscope. Mineral precipitates in the sampled culture were dissolved using oxalate before DAPI-staining. A 500 μL sample was collected anaerobically and an equal volume of marine TPE solution (100 mM Tris pH 7.0, 10 mM EDTA, and 300 mM sodium phosphate) was added without significantly changing the osmolality. Filter-sterilized oxalate solution (200 mM ammonium oxalate, and 120 mM oxalic acid) was added until the culture turns yellow. Cells in the mixture were collected on a membrane filter (pore size; 0.2 μm, Millipore). The effect of the oxalate treatment on the microbial cell counts was evaluated by counting cells from H_2_ grown culture with or without the treatment and confirmed to be negligible (data not shown).

### Electron equivalents (eeq) calculations

For direct comparison of the electrons donated by Fe^0^, all results are presented in millimolar electron equivalents (mM eeq). Electron equivalents were calculated based on the stoichiometry of the following reactions:1 mM H_2_ corresponds to 2 mM eeq (2H^+^ + 2e^−^ → H_2_), 1 mM CH_4_ corresponds to 8 mM eeq (CO_2_ + 8H^+^ + 8e^−^ → CH_4_ + 2H_2_O), 1 mM Fe^2+^ corresponds to 2 mM eeq (Fe^0^ → Fe^2+^ + 2e^−^). Total electron recoveries were calculated at the end of the incubation period (day 42) by summing the electron equivalents recovered as methane and hydrogen.

### Whole-cell cyclic voltammetry on carbon electrode

A two-chambered reactor with 300 mL volume (150 mL × 2) was filled with 200 mL modified NBRC medium 927 without Fe^0^ as the electrolyte. Graphite electrodes (75 × 25 × 10 mm) were used as the working and counter electrodes. The electrode was fixed to a titanium wire using a conductive epoxy (EPO-TEK H20E, EPOXY TECHNOLOGY), and then the connection part was covered with non-conductive epoxy (EPO-TEK 730, EPOXY TECHNOLOGY). A leak-free Ag/AgCl (3.4 M KCl) electrode was used as the reference electrode. Cyclic voltammetry was performed using a MultiEmStat3+ potentiostat (PalmSens BV, Houten, The Netherlands) with a scan rage of -1.2 to 0.2 V (vs. Ag/AgCl 3.4 M KCl) and a scan rate of 1 mV/s.

### Helium-ion microscopy

Strain Mic1c10 was incubated in 5 mL of modified NBRC medium 927 with an Fe^0^ foil (99.5%, 5 × 5 × 0.127 mm, Thermo Scientific Chemicals) in a 20 mL serum vial for 10 days. The foil was then fixed using 2% glutaraldehyde in marine TPE solution for 72 h. The specimen was washed three times with the marine TPE solution, dehydrated in different ethanol dilutions (50, 70, 80, 90, 95, and 100% × 3 times, 10 min each) and hexamethyldisilazane (30 s), and dried under a continuous flow of N_2_. All procedures were done anoxically in the serum vial.

Microbial cells were imaged using a Zeiss ORION NanoFAB Helium Ion Microscope with SE detection (Zeiss, Germany). He+ imaging was performed at 25 keV beam energy, with a probe current ranging from 0.08 to 0.01 pA, and a scan dwell time of 1 µs. Charge compensation was applied, if necessary, using a low-energy electron beam, a flood gun with 433 eV. Sample working distance is 8.7 mm.

### Genome sequencing and taxonomic analysis

The whole genome of the strain Mic1c10 was sequenced through Illumina MiSeq paired-end library technique by FASMAC Co. Ltd. (Japan). The genome sequence was assembled using SPAdes (v. 3.15.3)^27^ with iterated k-value 21, 33, 55, 77, 99, and 127. All the scaffolds were annotated using Rapid prokaryotic genome annotation system (PROKKA)^28^ and BlastKOALA (ver. 2.2)^29^. To resolve the taxonomy of Mic1c10, the sequence of the isolate was compared to the Type Strain Genome Server (TYGS) based on 16S rRNA gene and whole genome sequence.

### Phylogenomic analysis of Mic1c10

The genome of Mic1c10 was compared with multiple genomes (3 corrosive and 3 non-corrosive strains) of *M. maripaludis* for the Average nucleotide identity (ANI). The ANI values (all vs all) were computed using an alignment-free fastANI v1.33^30^ algorithm to identify the closest genome by orthologous mappings and alignment identity estimates. The similarity matrix was used to generate a heatmap using ggplot2 in R version 3.6.3^31^.

All seven genomes of *M. maripaludis*, including the newly sequenced Mic1c10, were annotated using PROKKA and subsequently used to construct the pangenome using ROARY (Rapid large-scale prokaryote pan-genome analysis)^32^. In brief, paralogous genes were excluded from the pan-genome analysis. Orthologous gene clusters were defined as groups of genes with a minimum of 95% sequence identity. The core genome was defined by the 447 genes present in at least 99% of the strains. Core genome multilocus sequence typing (cgMLST)^33^ was performed on all the 447 core genome genes identified across all strains. These sequences were concatenated, aligned, and a phylogenetic tree was constructed using the maximum likelihood algorithm in PHYLIP^34^. The resulting tree was visualized with FigTree v1.4.4 (http://tree.bio.ed.ac.uk/software/figtree/).

### Identification of the genomic islands

The genomes of corrosive and non-corrosive strains were compared to identify the presence of gene islands specific to all corrosive strains and gene islands specific to Mic1c10. These comparisons were visualized using MAUVE (Multiple alignment of conserved genomic sequence with rearrangement)^35^ and BRIG (Blast Ring Image generator)^35^. The genomic feature alignment was conducted using the progressive MAUVE tool with higher seed weight and full alignment. The selected genomes of corrosive and non-corrosive strains were further compared with BRIG to identify unique or distinct genomic regions among the strains. In BRIG, Mic1c10 was used as reference genome. For gene-island comparisons between strains, the genes on the islands were additionally analyzed using both BLASTn and BLASTp.

### Other bioinformatic analyses

To predict the protein localization, protein structure, protein-protein interactions, and potential glycosylation sites, all genes encoded on the genomic islands were analyzed using several tools with default parameters: InterPro^36^, DeepLocPro 1.0 for Archaea^37^, ColabFold^38^, PEPPI^39^, and NetNGlyc 1.0^40^. The type of signal peptides were identified using SignalP 5.0 for Archaea^41^.

To determine how the gene islands emerged, we screened for ‘alien’ genetic material and investigated the evolutionary roots of all the genes present. All tools were used with default parameters, unless specified otherwise. Putative HGT sites were identified using Alien_Hunter^42^, while mobile genetic elements were detected through MobileOGdb^43^. The evolutionary origins of the genes on these islands were further explored by identifying by PSI-BLAST and identifying orthologous groups using OrthoDB^44^ and EggNOG 5.0. Evolutionary rates were automatically calculated in OrthoDB^45^.

### Figure assembly

Composite figures were compiled using Biorender (https://www.biorender.com).

## Supplementary information

Supplementary File

## Data Availability

All raw sequence data for *M. maripaludis* strain Mic1c10 have been deposited at NCBI under accession numbers: BAAABJ010000001-BAAABJ010000038, the genome assembly accession number is ASM4036218v1 and the BioProject number is PRJDB9712. Other accession numbers for genome assemblies used for comparative genomics are: ASM82853v1 for *M. maripaludis* strain KA1, ASM82855v1 for *M. maripaludis* strain OS7, ASM1570936v1 for the environmental MAG MIC098Bin5, ASM1158v1 for *M. maripaludis* strain S2, ASM294532v1 for *M. maripaludis* strain DSM 2067 (also known as strain J.J.), and ASM1612v1 for *M. maripaludis* strain C5. All other data are included in the manuscript figures and supplementary materials or available from the corresponding authors upon reasonable request.
