# Supplementary material for: Adaptation of a methanogen to Fe0 corrosion via direct contact
Source: NPJ Biofilms Microbiomes. 2024 Oct 4;10:100. doi: 10.1038/s41522-024-00574-w (PMC11452385; doi:10.1038/s41522-024-00574-w)
Supplement: Supplementary file 1 — Supplementary File [file 41522_2024_574_MOESM1_ESM.pdf]

Supplementary note

Table of Contents

Supplementary Fig.1. H_2 partial pressures on Fe^0 for corrosive Mic1c10 vs. non-corossive JJ.

Supplementary Fig.2. Spent filtrate experiments with Mic1c10 2-weeks-old cell filtrate.

Supplementary Fig.3. Cyclic voltammograms of cells and spent filtrate media.

Supplementary Fig.4. Alginate experiments with strain Mic1c10.

Supplementary Fig.5. Pangenome analyses of Mic1c10 against other six *M. maripaludis* strains.

Supplementary Fig.6. Average nucleotide identity of Mic1c10 vs. other six strains.

Supplementary Fig.7. Architecture of gene islands specific to all corroders or unique to Mic1c10.

Supplementary Fig.8. Glycosylation sites and dimer formation of Mic1c10's [NiFe]-hydrogenase.

Supplementary Fig.9. Horizontal gene transfer and mobile genetic elements in all 7 genomes.

Supplementary Table 1. Tafel analysis determining the corrosion rate of Fe^0 by Mic1c10.

Supplementary Table 2. Electron recovery in products (CH_4 and H_2) by Mic1c10 grown on Fe^0.

Supplementary Table 3. Genome features of *Methanococcus maripaludis* strain Mic1c10

Supplementary Table 4. List of synteny gene clusters specific to corrosive strains.

Supplementary Table 5. List of synteny gene clusters unique to Mic1c10.

Supplementary Table 6. Surface proteins prone to N-glycosylation.

Supplementary Table 7. List of glycosyltransferases annotated in all seven strains.

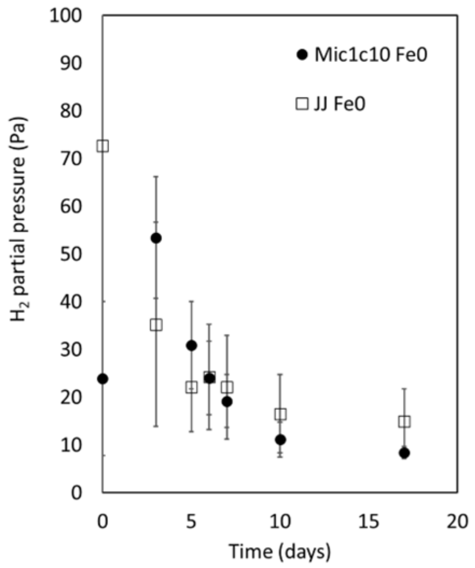

Supplementary Figure 1. Change in the H_2 partial pressure (Pa) over time (days) for two different *M. maripaludis* strains - corrosive Mic1c10 and non-corrosive JJ grown on Fe^0 (as explained below). These results show that the corrosive strain is as effective at consuming H_2 from Fe^0 as the non-corrosive strain, in direct contradiction to the cathodic depolarization theory.

Strain JJ (DSM 2067) was routinely cultivated in the modified NBRC medium 927 without Fe^0-granules but with H_2/CO_2 (80/20) as the electron and carbon sources. The experiment was initiated by inoculating 10% (v/v) culture of each strain to the modified NBRC medium 927 (5g Fe^0-granule in 50 mL medium). The pressure within each vial was monitored using a differential pressure manometer (RS-8890G, RS PRO), while the gas content was measured using the GC equipped with TCD. The incubation and measurements were extended until the vial got over-pressurized, indicating that the rate of H_2 evolution surpassed H_2 consumption. The experiment was conducted in triplicate, with the final data point for Mic1c10-Fe^0 being derived from a duplicate measurement.

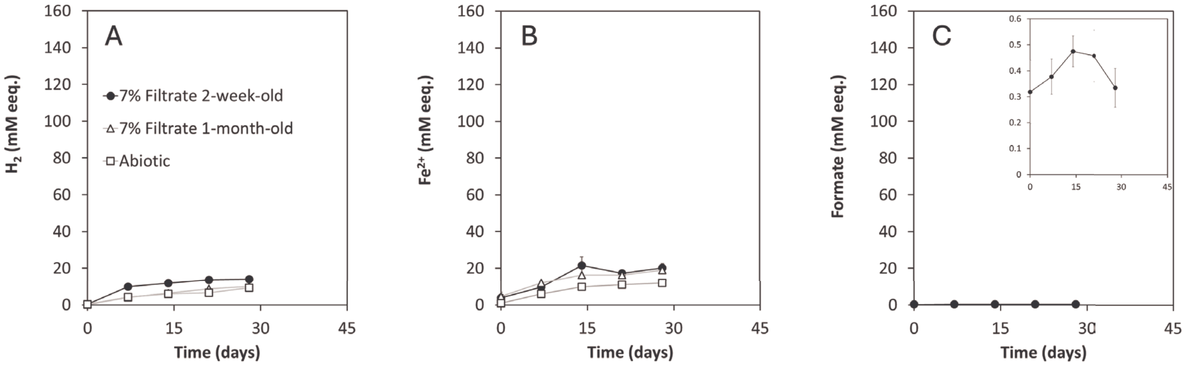

Supplementary Figure 2. Incubations of Fe^0 with 2-weeks old cell-filtrate (black circles) compared to 1-month old cell filtrate (empty triangles) and abiotic controls (empty squares). Measured over time were (A) hydrogen gas, (B) ferrous iron (Fe^{2+}), and (C) formate. Formate measurements in the 2-weeks-old filtrate incubations were taken through the incubation. In the 1-month-old incubations, formate was only measured at the end of the incubation (day 42) reaching a concentration of 0.16 ± 0.04 mM (0.32 ± 0.08 mM eeq; n ≥ 3) and is therefore not shown in the figure. Formate in abiotic Fe^0-media controls formate was consistently below the detection limit in measurements independent of these experiments, so it is also not shown in the figure. The inset shows a magnified Y-axis. All incubations were carried out in triplicate (n ≥ 3). Methane could not be detected in any of these incubations.

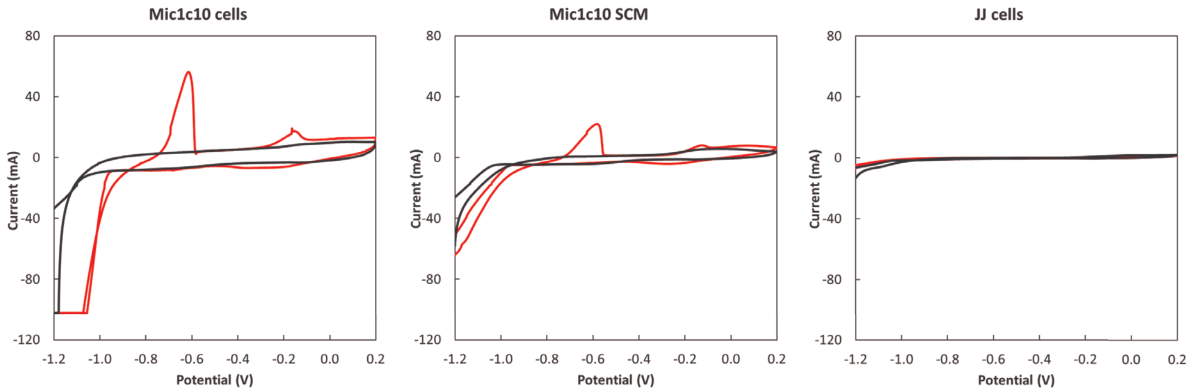

Supplementary Figure 3. Cyclic voltammograms of the culture of strain Mic1c10 grown on Fe^0 (A) and its spent cell media/SCM (B) and that of the non-corrosive strain JJ cells (C) (red traces). Voltammograms of the blank medium in each reactor are shown in black traces. The scan rate of the cyclic voltammetry (CV) was 1 mV/s.

A two-chambered reactor with 300 mL volume (150 mL × 2) was filled with 200 mL modified NBRC medium 927 without Fe^0 granules, as the electrolyte. Graphite electrodes (75 × 25 × 10 mm) were used as the working and counter electrodes. The electrode was fixed onto a titanium wire using the conductive epoxy, and then the connection part was covered with the non-conductive epoxy. A leak-free Ag/AgCl (3.4 M KCl) electrode was used as the reference electrode. CV was performed using a MultiEmStat3+ potentiostat (PalmSens BV, Houten, The Netherlands) with a scan range of -1.2 to 0.2 V (vs. Ag/AgCl 3.4 M KCl). CV on a blank medium was measured first, and then 50% of the medium was replaced with the inoculum (Mic1c10, SCM, or JJ).

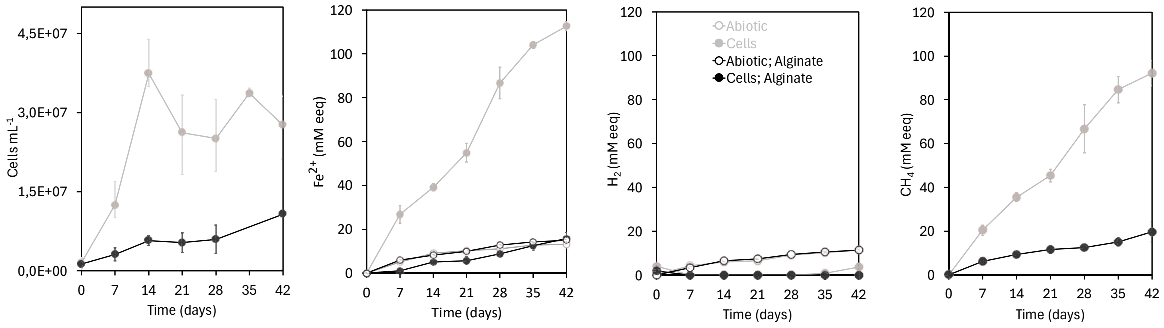

Supplementary Figure 4. Incubations with Fe^0 coated with alginate. Gasses, like H_2 cross the alginate barrier, so it is expected that alginate-coated-Fe^0 could still provide abiotic H_2 to the cells. (A) Growth of cells with alginate-coated Fe^0 (black circles) versus cells grown on Fe^0-directly (gray circles); (B) Formation of ferrous iron (Fe^{2+}), (C) hydrogen and (D) methane by cells grown on alginate-coated Fe^0 (black circles) versus Fe^0-directly (gray circles), and abiotic buildup of corrosion products (Fe^{2+}, H_2, CH_4) on alginate-coated Fe^0 (empty circles). The absence of the abiotic symbols in the graphs signifies that CH_4 and cells could not be detected at all through the incubation. All incubations were carried out at a minimum in triplicate (n≥3).

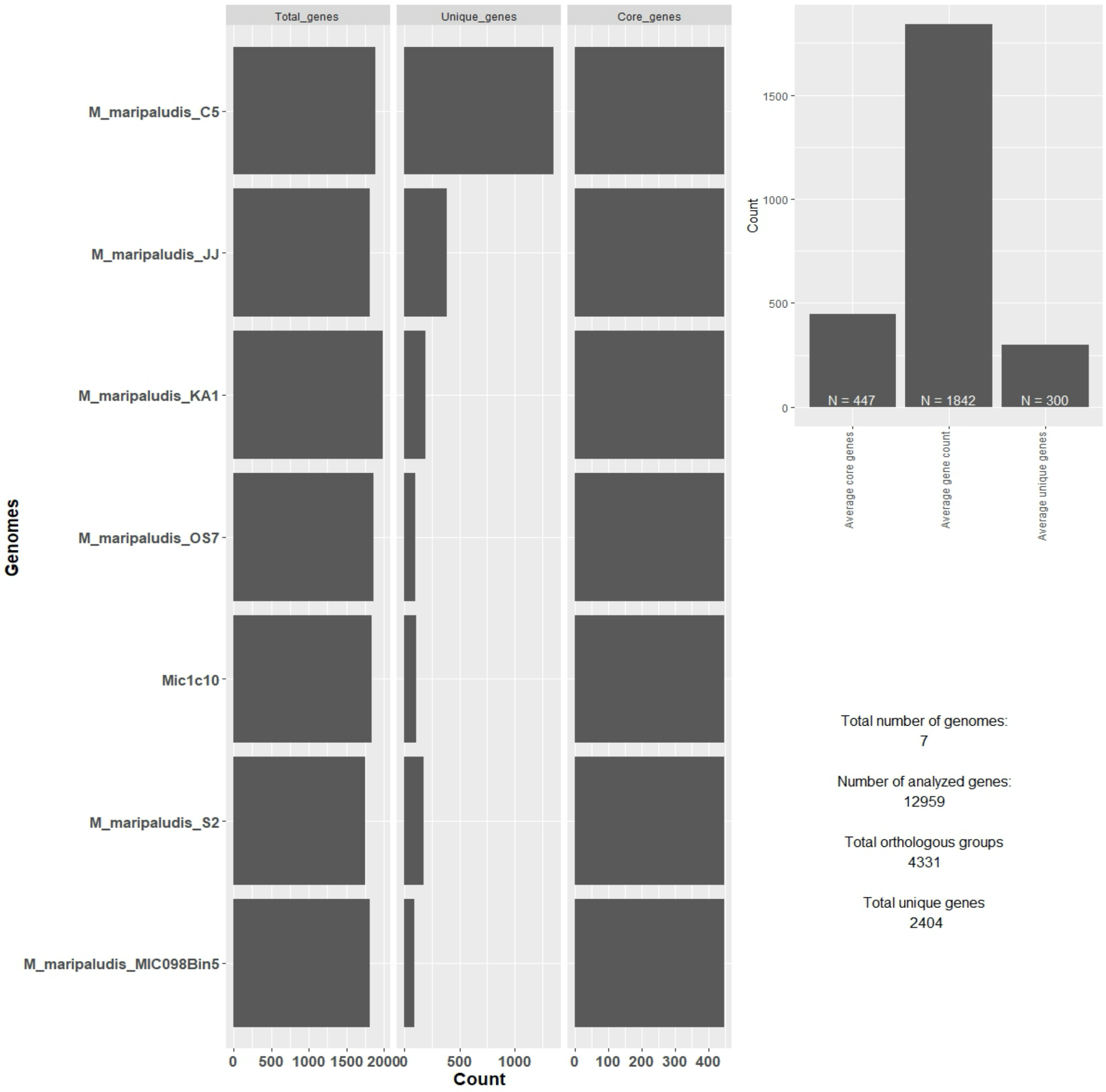

Supplementary Figure 5. Pangenome analyses of seven *Methanococcus maripaludis* strains including strain Mic1c10. The core genome includes 447 genes with 99-100% sequence identity between all strains. Strain C5 was found to have most unique genes among the selected strains. Unique genes to a strain were absent from the genomes of all the other strains. A total of 12959 genes were analyzed with an average gene count per genome of 1842 genes.

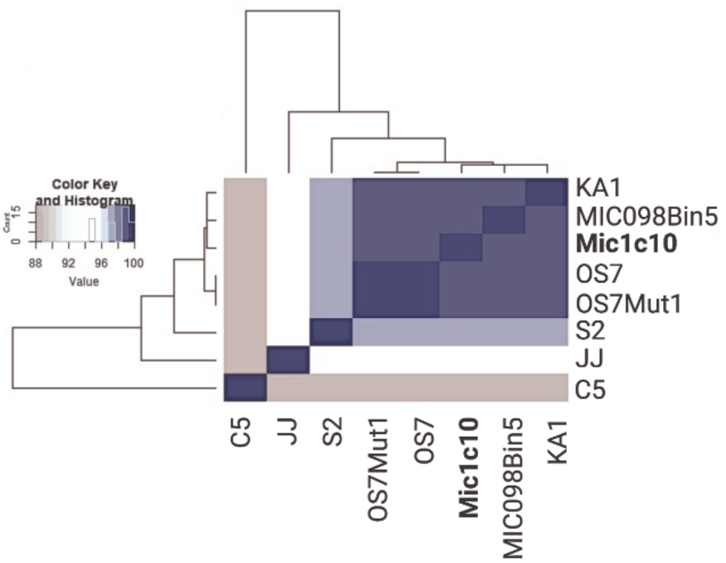

Supplementary Figure 6. Average nucleotide identity of Mic1c10 against 3 corrosive strains (KA1, MIC098Bin05, OS7) and 3 strains of *M. maripaludis* that have not been associated with corrosion (S2, JJ, C5).

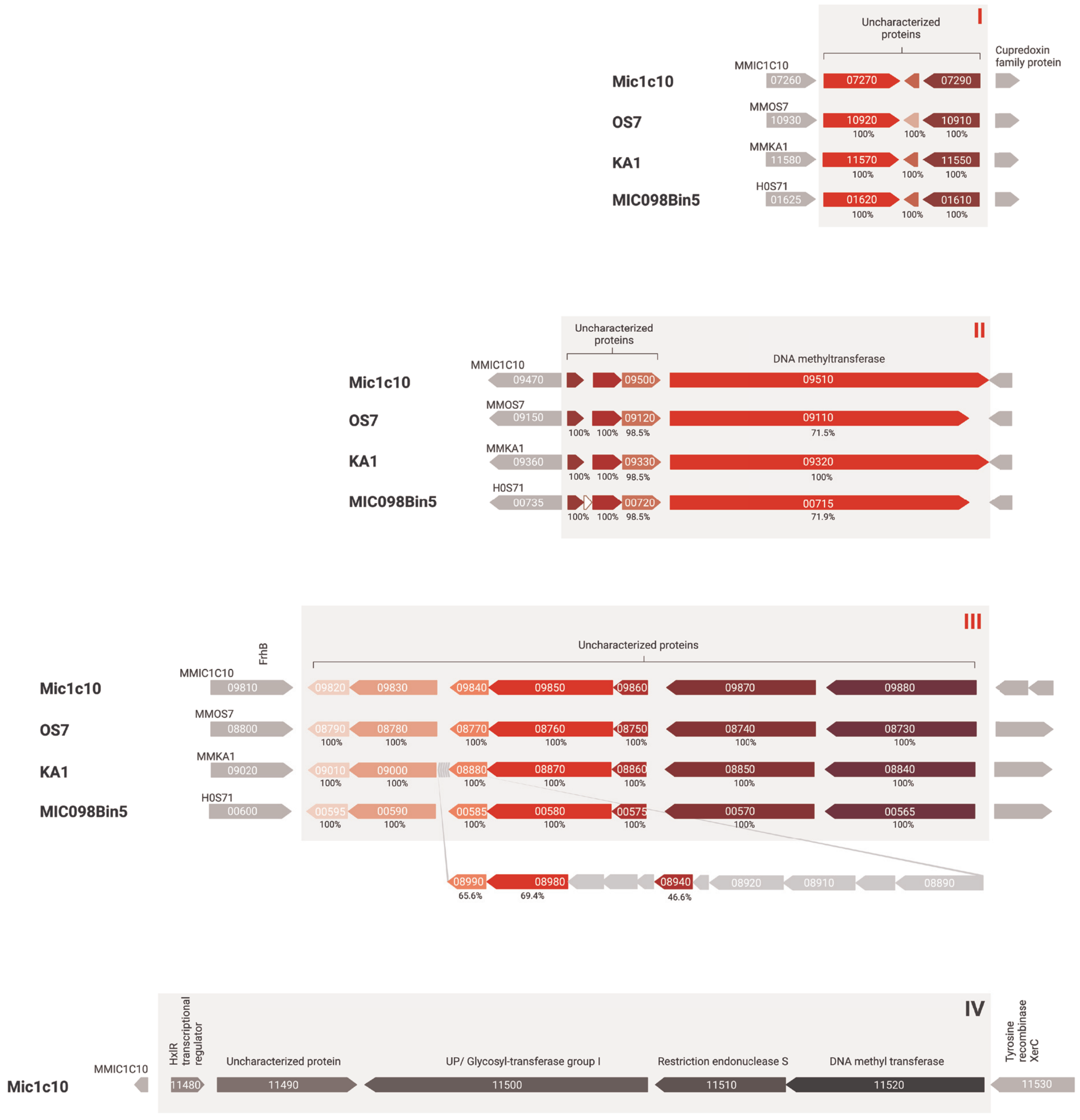
I
Uncharacterized proteins
Cupredoxin family protein
Mic1c10
MMIC1C10
07260
07270
07290
OS7
MMOS7
10930
10920
10910
100%
100%
100%
KA1
MMKA1
11580
11570
11550
100%
100%
100%
MIC098Bin5
H0S71
01625
01620
01610
100%
100%
100%
II
Uncharacterized proteins
DNA methyltransferase
Mic1c10
MMIC1C10
09470
09500
09510
OS7
MMOS7
09150
09120
09110
100% 100% 98.5%
71.5%
KA1
MMKA1
09360
09330
09320
100% 100% 98.5%
100%
MIC098Bin5
H0S71
00735
00720
00715
100% 100% 98.5%
71.9%
III
FrhB
Uncharacterized proteins
Mic1c10
MMIC1C10
09810
09820
09830
09840
09850
09860
09870
09880
OS7
MMOS7
08800
08790
08780
08770
08760
08750
08740
08730
100% 100% 100% 100% 100% 100% 100%
KA1
MMKA1
09020
09010
09000
08880
08870
08860
08850
08840
100% 100% 100% 100% 100% 100% 100%
MIC098Bin5
H0S71
00600
00595
00590
00585
00580
00575
00570
00565
100% 100% 100% 100% 100% 100% 100%
08990
08980
08940
08920
08910
08890
65.6%
69.4%
46.6%
IV
Mic1c10
MMIC1C10
HxlR transcriptional regulator
Uncharacterized protein
UP/ Glycosyl-transferase group I
Restriction endonuclease S
DNA methyl transferase
Tyrosine recombinase XerC
11480
11490
11500
11510
11520
11530

cont.1/2

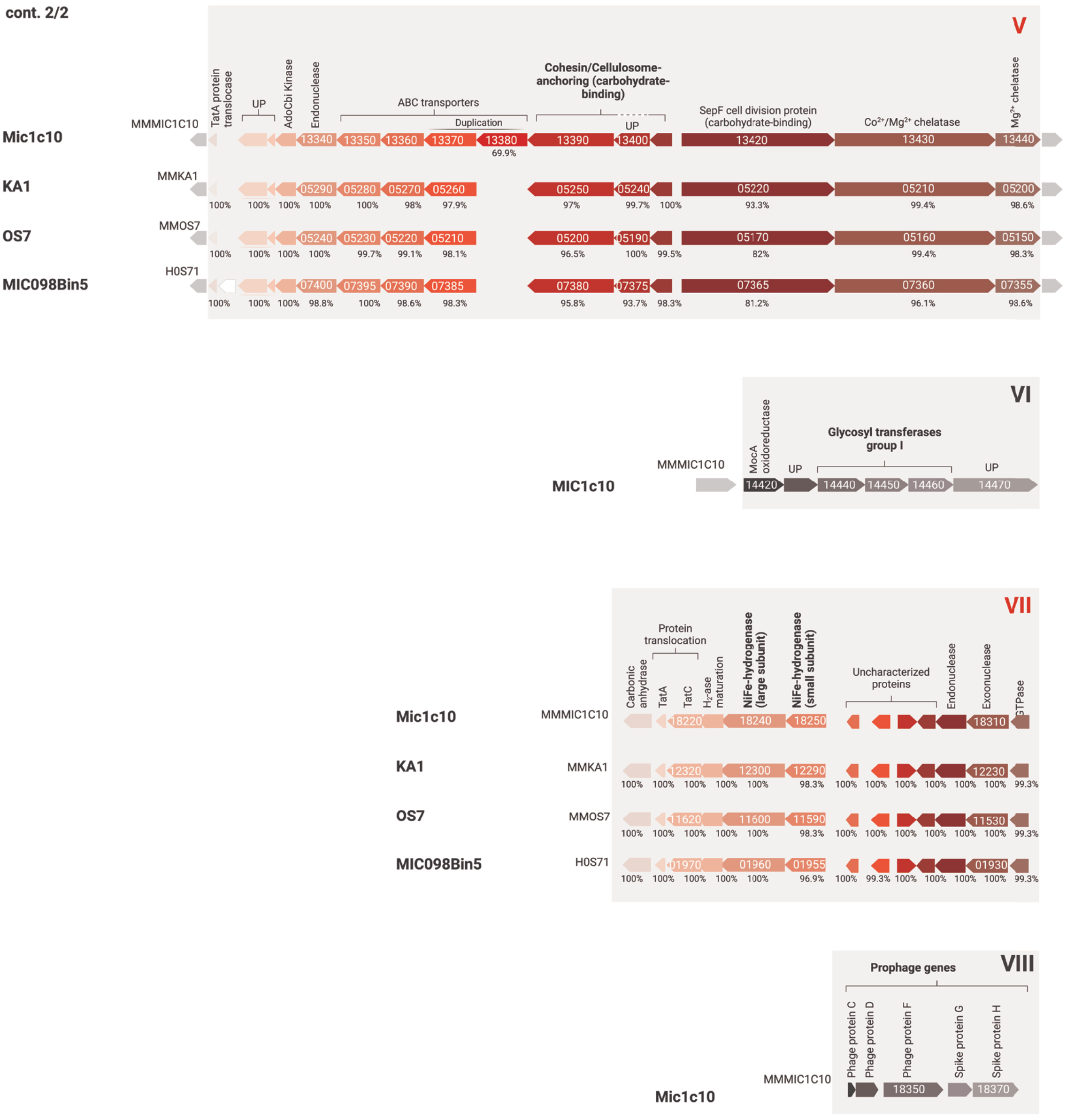

Supplementary Figure 7. Gene clusters specific to corrosive strains (I, II, III, V, VII) or unique to Mic1c10 (IV, VI, VIII). The figure is split on two pages.

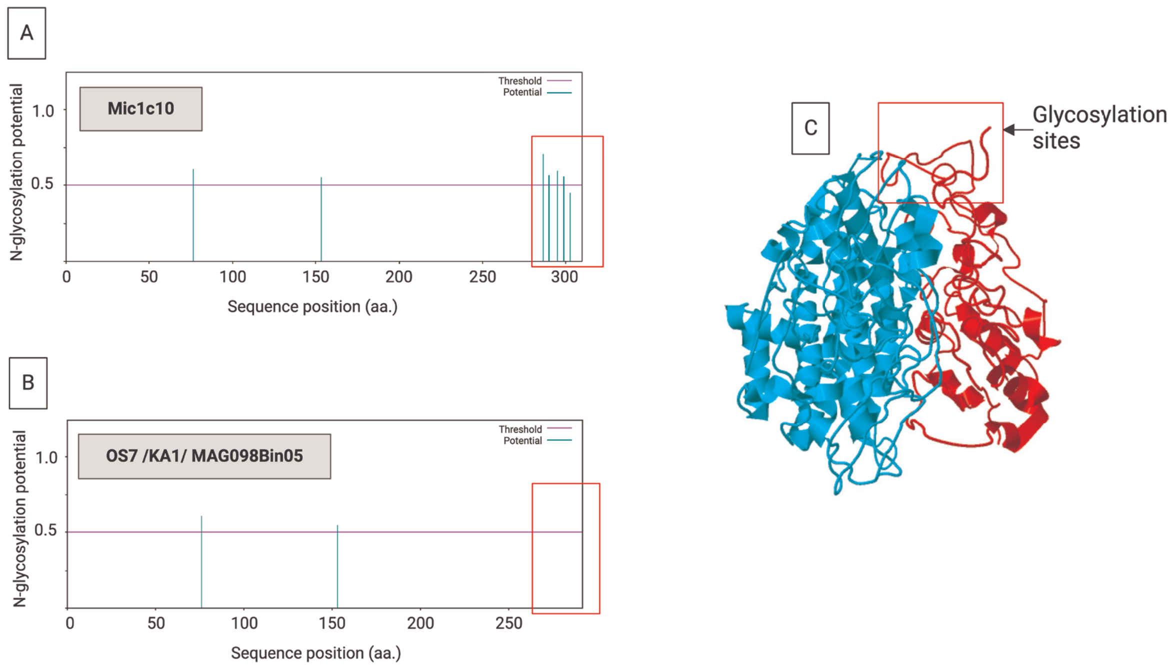

Supplementary Figure 8. N-linked glycosylation sites present on the small subunit (SSU) of the MIC-island [NiFe]-hydrogenases of strains (A) Mic1c10, (B) OS7, KA1 and MAG098Bin05. (C) The predicted dimer structure of the hydrogenase of Mic1c10. The C-terminal end of the SSU, where four N-linked glycosylation sites unique to Mic1c10 are located, is highlighted. NetNGlyc 1.0 was used to detect the potential N-linked glycosylation sites, and PEPPI was used to predict the interaction between the two subunits of the MIC hydrogenase of Mic1c10.

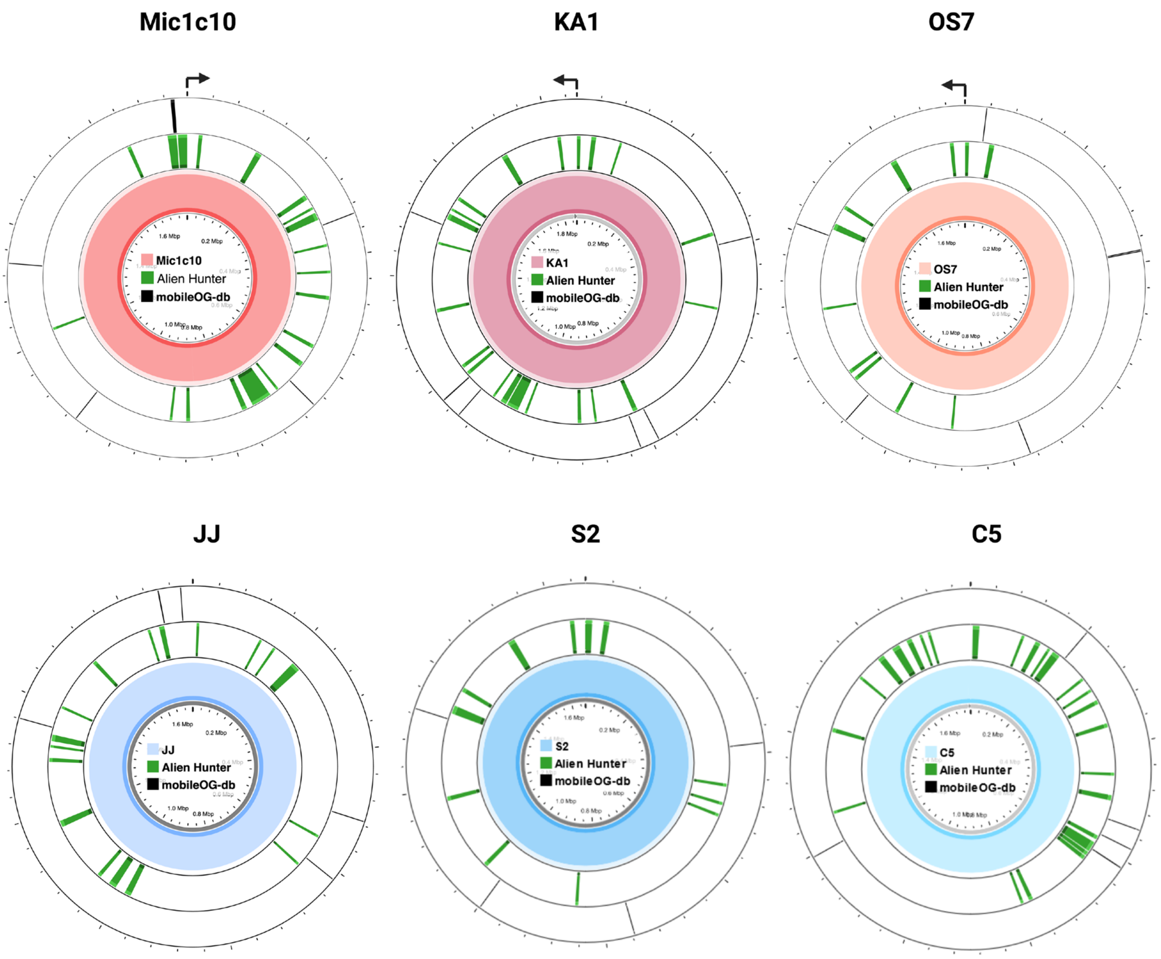

Supplementary Figure 9. Horizontal gene transfer (HGT) regions and mobile genetic elements (MGE) in corrosive strains (Mic1c10, KA1, OS7) versus non-corrosive strains (JJ, S2 and C5). HGT-regions were identified using the bioinformatic tool called Alien Hunter (in green, intermediate circles), which detects nucleotide regions with signatures different from the host genome by analyzing factors such as codon usage, GC content, and oligonucleotide frequencies. MGEs were identified using mobileOG-db (in black, outer circles), a bioinformatics resource that classifies mobile genetic elements (MGEs) including entities like plasmids, transposons, integrons, and prophages.

Supplementary Table 1. Tafel parameters of carbon-steel sheets exposed for 2 weeks to intact or heat-killed cells of strain Mic1c10. The average and standard deviation of triplicate reactors for each condition are shown. A detailed explanation of the experiment is presented below.

Electrochemical measurements of corrosion activity were conducted using a three-electrode single-cambered electrochemical reactor with a volume of 1 L filled with 500 mL culture medium. A carbon felt and an Ag/AgCl (saturated KCl) were used as counter and reference electrodes, respectively. The working electrode was prepared by polishing a mild steel coupon (25 x 75 x 1 mm) to a 1,200-grit finish. Subsequently, the coupon was fixed to a titanium wire with conductive epoxy (EPO-TEK H20E, EPOXY TECHNOLOGY). Following drying, the connection part was completely covered with non-conductive epoxy (EPO-TEK 730, EPOXY TECHNOLOGY), and then the working electrode was sterilized with 96% (v/v) ethanol. The reactor was maintained under open-circuit conditions at room temperature to facilitate corrosion measurement. Linear sweep voltammetry (LSV) was periodically performed using an AMEL2553 potentiostat/galvanostat (Amel Electrochemistry, Milan, Italy) with a scan rate of 0.167 mV/s. The initial scan range was established from -800mV to -500mV and adjusted to hold the corrosion potential within the scan range. The resultant current-potential (I-V) curve was analyzed using a Tafel fitting tool in CView ver. 3.5a (Scribner Associates Inc. NC, USA). This facilitated the calculation of corrosion parameters, including corrosion current (I_{corr}) and corrosion potential (E_{corr}). The corrosion current density (J_{corr}) was further derived from the I_{corr} and the known surface area of the steel coupon (39.5 cm^2).

Parameter	**Mic1c10 intact cells**	**Mic1c10 heat-killed cells**
**J_{corr} (μA/cm^2)**	186.7 ± 138.1	1.3 ± 1.0
**E_{corr} (mV)**	-594.3 ± 13.4	-657.9 ± 17.0
**b_a (mV)**	177.3 ± 54.3	44.7 ± 12.3
**b_c (mV)**	625.8 ± 167.0	86.0 ± 23.7

Supplementary Table 2. Electron recovery in products (CH_4 and H_2) by *Methanococcus maripaludis* strain Mic1c10 provided with Fe^0 as sole electron donor. Comparison to the accumulation of Fe^{2+} as a byproduct of Fe^0 oxidation. These data rely on initial (day 0) and final (day 42) data points of growth curves.

**Treatment**	**Accumulated H_2 (mM eeq)** $[H_2]_f$-$[H_2]_i$ = mM $H_2 \times 2e^-$	**Accumulated CH_4 (mM eeq)** $[CH_4]_f$-$[CH_4]_i$ = mM $CH_4 \times 8e^-$	**Accumulated Fe^{2+} (mM eeq)** $[Fe^{2+}]_f$-$[Fe^{2+}]_i$ = mM $Fe^{2+} \times 2e^-$
Abiotic	11.1 ± 0.8	0.0 ± 0.0	13.1 ± 1.0
Active cells	-0.3 ± 1.6	92.2 ± 5.8	112.7 ± 2.2
Filtrate 7%	13.3 ± 0.9	0.0 ± 0.0	13.9 ± 2.8
Filtrate 50%	12.3 ± 0.7	0.1 ± 0.0	-27.5 ± 5.3
Active cells, alginate-Fe^0	-1.9 ± 0.5	19.7 ± 4.6	15.7 ± 5.0
Abiotic, alginate-Fe^0	11.0 ± 0.8	0.0 ± 0.0	14.7 ± 0.7

Supplementary Table 3. Genome features of *Methanococcus maripaludis* strain Mic1c10.

**Attribute**	**Value**
Genome size (bp)	**1755831**
Genome coverage	**1099x**
Coding sequences	**1846**
GC content (%)	**33.1**
Number of contigs	**38**
tRNAs	**36**
rRNAs (5S, 16S, 23S)	**4**

Supplementary Table 4. Synteny gene clusters specific to corrosive strains.

Threshold of protein identity >60% aminoacid sequence identity (aa. ID %) and >80% subject coverage. BT indicates values below threshold, dash indicates absence. EggNOG abbreviations: A – Archaeal ancestry, B – Bacterial ancestry, R – Root. Evolutionary rates from OrthoDB. Membrane helices from Interpro. Signal peptide (SignalP 5.0) abbreviations: SP – standard signal peptides, LIPO – lipoprotein signal peptides, and TAT – TAT signal peptides. Cellular localization from DeepLocPro 1.0 abbreviations: C – cytoplasmic, CW – cell wall, CM – cell membrane, E – extracellular.

Position I, II, III, V and VII correspond to the labels shown in Fig. 2a.

Position	Annotation	EggNOG orthologous group	Evolutionary rate	Phobius TM	TMHMM	Signal P/LIPO/TAT	Localization DeepLoc	Mic1c10	N-X-S/T motif	KA1	AA ID %	N-X-S/T motif	OS7	AA ID %	N-X-S/T motif	MIC098Bin05	AA ID %	N-X-S/T motif	JJ	AA ID %	N-X-S/T motif	S2	AA ID %	N-X-S/T motif	C5	AA ID %	N-X-S/T motif
	Hypothetical protein (cell wall binding repeat 2-domain/anchoring of enzymes)	A; E (arCOG00388)	1,4	-	-	SP	**E**	MMMIC1C10_07270	2	MMKA1_11570	100	2	MMOS7_10920	100	2	H0S71_01620	100	2	BT	-	-	BT	-	-	BT	-	-
I	Hypothetical protein (VWA2-domain/protein-protein interactions, cell adhesion)	-	-	-	-	-	**E**	MMMIC1C10_07280	-	MMKA1_11560	100	-	MMOS7_RS05560	100	-	H0S71_01615	100	-	-	-	-	BT	-	-	BT	-	-
	Non-cytoplasmic hypothetical protein	-	-	-	-	SP	**E**	MMMIC1C10_07290	3	MMKA1_11550	100	3	MMOS7_10910	100	3	H0S71_01610	100	3	-	-	-	-	-	-	-	-	-
	Hypothetical protein (Zn-finger domain)	A (arCOG09534)	1,1	-	-	-	C	MMMIC1C10_09480	-	MMKA1_09350	100	-	MMOS7_09140	100	-	H0S71_00730	100	-	BT	-	-	-	-	-	BT	-	-
II	Hypothetical protein	A (arCOG06614)	1,5	-	-	-	C	MMMIC1C10_09490	-	MMKA1_09340	100	-	MMOS7_09130	100	-	H0S71_00725	100	-	-	-	-	BT	-	-	-	-	-
	Hypothetical protein (DUF2283-domain)	A (arCOG05051)	0,9	-	-	-	C	MMMIC1C10_09500	1	MMKA1_09330	99	1	MMOS7_09120	99	1	H0S71_00720	99	1	-	-	-	BT	-	-	-	-	-
	Site specific DNA methyltransferase (adenine specific)	R (COG1002)	-	-	-	-	C	MMMIC1C10_09510	2	MMKA1_09320	100	2	MMOS7_09110	72	2	H0S71_00715	72	2	BT	-	-	BT	-	-	BT	-	-
	Hypothetical protein	B (ENOG5033BB1)	1,0	-	-	-	C	MMMIC1C10_09820	-	MMKA1_09010	100	-	MMOS7_08790	100	-	H0S71_00595	100	-	-	-	-	-	-	-	-	-	-
	Hypothetical protein (RHS-repeat, ligand binding/traficking, secretion, transport)	R (COG3209)	1,1	-	-	-	**E**	MMMIC1C10_09830	-	MMKA1_09000	100	-	MMOS7_08780	100	-	H0S71_00590	100	-	-	-	-	-	-	-	-	-	-
	Hypothetical protein	A (arCOG12033)	-	2	3	(SP)	**CM**	MMMIC1C10_09840	1	MMKA1_08880	100	1	MMOS7_08770	100	1	H0S71_00585	100	1	-	-	-	-	-	-	MmarC5_0526	82	-
III	Hypothetical protein (RHS-repeat, ligand binding/traficking, secretion, transport)	R (COG3209)	-	2	2	-	**E**	MMMIC1C10_09850	5	MMKA1_08870	100	5	MMOS7_08760	100	5	H0S71_00580	100	5	-	-	-	-	-	-	-	-	-
	Hypothetical protein (phosphohydrolase)	A (arCOG11993)	-	3	3	-	**CM**	MMMIC1C10_09860	-	MMKA1_08860	100	-	MMOS7_08750	100	-	H0S71_00575	100	-	-	-	-	-	-	-	MmarC5_1114	68	-
	Hypothetical protein (PINc/GNAT domain; exonuclease/acetyltransferase)	A (arCOG06636)	1,1	-	-	-	C	MMMIC1C10_09870	2	MMKA1_08850	100	2	MMOS7_08740	100	2	H0S71_00570	100	2	BT	-	-	BT	-	-	BT	-	-
	Radical SAM-domain containing dimethyltransferase (replication/recombination/repair)	R (COG1964)	1,0	-	-	-	**CM**	MMMIC1C10_09880	1	MMKA1_08840	100	1	MMOS7_08730	100	1	H0S71_00565	100	1	BT	-	-	BT	-	-	BT	-	-
	Sec-independent protein translocase protein TatA	-	-	-	-	-	**E**	MMMIC1C10_13300	-	MMKA1_RS02745	100	-	MMOS7_RS09360	100	-	H0S71_07425	100	-	-	-	-	BT	-	-	-	-	-
	Hypothetical protein	-	-	-	-	-	**E**	MMMIC1C10_13310	3	MMKA1_05320	100	3	MMOS7_05270	100	3	H0S71_07415	100	3	-	-	-	BT	-	-	-	-	-
	MarR-type HTH domain-containing protein (DNA binding/transcriptional regulator)	A (arCOG01057)	1,5	-	-	-	C	MMMIC1C10_13320	1	MMKA1_05310	100	1	MMOS7_05260	100	1	H0S71_07410	100	1	BT	-	-	BT	-	-	BT	-	-
	Adenosylcobinamide kinase/adenosylcobinamide-phosphate guanylyltransferase (vit. B12 biosynthesis)	R (COG2087)	1,2	-	-	-	C	MMMIC1C10_13330	-	MMKA1_05300	100	-	MMOS7_05250	100	-	H0S71_07405	100	-	-	-	-	-	-	-	-	-	-
	TIM barrel protein (carbohydrate transport and metabolism)	R (COG0648)	1,2	-	-	-	C	MMMIC1C10_13340	3	MMKA1_05290	100	3	MMOS7_05240	100	3	H0S71_07400	99	3	BT	-	-	BT	-	-	BT	-	-
	ABC transporter, permease, BtuC-like (vit.B12/Fe3+ transport)	R (COG0609)	1,0	10	9	SP	**CM**	MMMIC1C10_13350	-	MMKA1_05280	100	-	MMOS7_05230	100	-	H0S71_07395	100	-	BT	-	-	BT	-	-	BT	-	-
	ABC transporter, permease, BtuC-like (vit.B12, Fe3+ transport)	R (COG0609)	1,0	-	9	(SP)	**CM**	MMMIC1C10_13360	1	MMKA1_05270	98	1	MMOS7_05220	99	1	H0S71_07390	99	1	BT	-	-	BT	-	-	BT	-	-
V	ABC transporter substrate-binding protein B12 /periplasmic	A (arCOG03417)	1,0	-	-	LIPO	**E**	MMMIC1C10_13370	-	MMKA1_05260	98	-	MMOS7_05210	98	-	H0S71_07385	98	-	BT	-	-	BT	-	-	-	-	-
	ABC transporter substrate-binding protein B12 /periplasmic	A (arCOG03417)	1,0	-	-	SP	**E**	MMMIC1C10_13380	-	MMKA1_05260	70	-	MMOS7_05210	70	-	H0S71_07385	69	-	BT	-	-	BT	-	-	-	-	-
	Cellulosome-anchoring protein (cohesin2 & PKD9-domains/carbohydrate-binding)	A (arCOG09552)	1,4	-	9	SP	**E**	MMMIC1C10_13390	25	MMKA1_05250	97	24	MMOS7_05200	97	23	H0S71_07380	96	21	-	-	-	-	-	-	-	-	-
	Hypothetical protein	A (arCOG09551)	1,5	1	1	SP	**CM**	MMMIC1C10_13400	4	MMKA1_05240	100	4	MMOS7_05190	100	4	H0S71_07375	94	4	-	-	-	-	-	-	-	-	-
	Cellulosome-anchoring protein (CBM2/CBM3-domain/carbohydrate-binding)	A (arCOG09552)	1,5	-	-	SP	**E**	MMMIC1C10_13410	1	MMKA1_05230	100	1	MMOS7_05180	99	1	H0S71_07370	98	1	-	-	-	-	-	-	-	-	-
	SepF-like cell division protein (cohesin2 & CARDB-domains/ carbohydrate-binding)	A (arCOG09552)	-	-	-	LIPO	**CW**	MMMIC1C10_13420	26	MMKA1_05220	93	30	MMOS7_05170	82	36	H0S71_07365	81	22	-	-	-	-	-	-	-	-	-
	Mg2+ chelatase CobN-type (vit B12/corinoid biosynthesis/coenzyme transport and metabolism)	R (COG1429)	1,2	-	-	-	C	MMMIC1C10_13430	6	MMKA1_05210	99	6	MMOS7_05160	99	6	H0S71_07360	96	6	-	-	-	BT	-	-	BT	-	-
	Mg2+-chelatase CHLI-like (vit B12/corinoid biosynthesis//coenzyme transport and metabolism)	R (COG1239)	1,0	-	-	-	C	MMMIC1C10_13440	1	MMKA1_05200	99	1	MMOS7_05150	98	1	H0S71_07355	99	1	BT	-	-	BT	-	-	BT	-	-
	Carbonic anhydrase (carbohydrate transport and metabolism)	R (COG0663)	1,0	-	1	SP	**CM**	MMMIC1C10_18200	2	MMKA1_12340	100	2	MMOS7_11640	100	2	H0S71_01980	100	2	BT	-	-	BT	-	-	BT	-	-
	Sec-independent protein translocase TatA	R (COG1826)	0,8	1	1	SP	**CM**	MMMIC1C10_18210	-	MMKA1_12330	100	-	MMOS7_11630	100	-	H0S71_01975	100	-	-	-	-	-	-	-	-	-	-
	Sec-independent protein translocase TatC (Intracellular trafficking, secretion & vesicular transport)	R (COG0805)	1,3	6	5	-	**CM**	MMMIC1C10_18220	-	MMKA1_12320	100	-	MMOS7_11620	100	-	H0S71_01970	100	-	-	-	-	-	-	-	-	-	-
	Hydrogenase maturation protease	R (COG0680)	1,1	-	-	-	C	MMMIC1C10_18230	-	MMKA1_12310	100	-	MMOS7_11610	100	-	H0S71_01965	100	-	BT	-	-	BT	-	-	BT	-	-
	Ni/Fe hydrogenase large subunit (NiFe) LSU	R (COG3259)	1,2	-	-	-	**E**	MMMIC1C10_18240	3	MMKA1_12300	100	3	MMOS7_11600	100	3	H0S71_01960	100	3	BT	-	-	BT	-	-	BT	-	-
	Ni/Fe hydrogenase small subunit (TAT signal, FeS) SSU	R (COG1941)	1,1	-	-	TAT	**E**	MMMIC1C10_18250	6	MMKA1_12290	98	2	MMOS7_11590	98	2	H0S71_01955	97	2	BT	-	-	BT	-	-	BT	-	-
VII	Hypothetical protein (non-cytoplasmatic domain)	B (ENOG5033NVI)	0,9	-	1	SP	**E**	MMMIC1C10_18260	7	MMKA1_12280	100	7	MMOS7_11580	100	7	H0S71_09260	100	7	BT	-	-	-	-	-	-	-	-
	Hypothetical protein (non-cytoplasmatic & FxLYD domains)	A (arCOG10172)	-	1	1	SP	**E**	MMMIC1C10_18270	2	MMKA1_12270	100	2	MMOS7_11570	100	2	H0S71_01950	99	2	BT	-	-	-	-	-	-	-	-
	Hypothetical protein (PINc-domain; endonuclease activity)	A (arCOG04116)	0,9	-	-	-	C	MMMIC1C10_18280	-	MMKA1_12260	100	-	MMOS7_11560	100	-	H0S71_01945	100	-	BT	-	-	BT	-	-	BT	-	-
	Hypothetical protein (cytoplasmic & FxLYD domains)	A (arCOG10172)	1,1	1	1	(SP)	**E**	MMMIC1C10_18290	-	MMKA1_12250	100	-	MMOS7_11550	100	-	H0S71_01940	100	-	-	-	-	-	-	-	-	-	-
	Endonuclease III (base-excision repair)	R (COG0177)	1,0	-	-	-	C	MMMIC1C10_18300	1	MMKA1_12240	100	1	MMOS7_11540	100	1	H0S71_01935	100	1	BT	-	-	BT	-	-	BT	-	-
	Restriction exonuclease V-like (replication, recombination & repair)	A (arCOG06102)	1,2	-	-	-	C	MMMIC1C10_18310	-	MMKA1_12230	100	-	MMOS7_11530	100	-	H0S71_01930	100	-	-	-	-	-	-	-	-	-	-
	Hypothetical GTPase (replication, recombination & repair)	R (COG2229)	1,1	-	-	-	C	MMMIC1C10_18320	-	MMKA1_12220	99	-	MMOS7_11520	99	-	H0S71_01925	99	-	BT	-	-	BT	-	-	BT	-	-

Supplementary Table 5. Unique genes and synteny gene clusters specific to strain Mic1c10.

Threshold of protein identity >60% aminoacid sequence identity (aa. ID %) and >80% subject coverage. BT indicates values below threshold, dash indicates absence. EggNOG abbreviations: A – Archaeal ancestry, B – Bacterial ancestry, R – Root. Evolutionary rates from OrthoDB. Membrane helices from Interpro. Signal peptide (SignalP 5.0) abbreviations: SP – standard signal peptides, LIPO – lipoprotein signal peptides, and TAT – TAT signal peptides. Cellular localization from DeepLocPro 1.0 abbreviations: C – cytoplasmic, CW – cell wall, CM – cell membrane, E – extracellular. Number of N-linked glycosylation sites (N-X-S/T) by NetNGlyc 1.0.

Positions IV, VI and VIII correspond to the labels shown in Fig. 2a.

Position	Annotation	EggNOG orthologous group	Evolutionary rate	Phobius TM	TMHMM	Signal peptide/LIPO/TAT	Localization	Mic1c10	N-X-S/T motif	KA1	OS7	MIC098	C5	S2	JJ
	Hypothetical protein	-	-	-	-	-	C	MMMIC1C10_03020	-	-	-	-	-	-	-
	Hypothetical protein (UDPGT domain/ glycosyltransferase-like)	-	-	1	1	-	C	MMMIC1C10_04660	-	-	-	-	-	-	-
	Hypothetical protein	-	-	-	3	(SP)	C	MMMIC1C10_09210	-	-	-	-	-	-	-
	Hypothetical protein	-	-	-	-	(SP)	C	MMMIC1C10_09980	1	-	-	-	-	-	-
	Hypothetical protein	A (arCOG05069)	1,2	-	-	-	C	MMMIC1C10_10800	-	BT	BT	BT	BT	BT	BT
	Ferrous iron transport protein B	R (COG0370)	1,2	10	9	-	**CM**	MMMIC1C10_10810	2	BT	BT	BT	BT	BT	BT
	Hypothetical protein	-	1,0	-	-	(SP)	**E**	MMMIC1C10_10820	1	-	-	BT	-	-	-
	Hypothetical protein	-	-	-	-	-	C	MMMIC1C10_11190	1	-	-	-	-	-	-
	Hypothetical protein (Myb/SANT-like DNA binding domain, typical of bt. transposon proteins)	B (ENOG50312H4)	1,0	-	-	-	C	MMMIC1C10_11200	1	-	-	-	-	-	-
	Hypothetical protein (integral component of the membrane, ek.)	-	1,6	-	-	-	C	MMMIC1C10_11210	-	-	-	-	-	-	-
	Hypothetical protein (AIG1-domains, resistance to bacteria)	-	-	-	-	-	C	MMMIC1C10_11220	2	-	-	-	-	-	-
	Hypothetical protein (WD40-repeats/assembly of protein complexes, ek.)	-	-	-	-	-	C	MMMIC1C10_11230	-	-	-	-	-	-	-
	Hypothetical protein	-	-	2	1	SP	**CM**	MMMIC1C10_11290	1	-	-	-	-	-	BT
	Hypothetical protein	-	-	2	2	-	**CM**	MMMIC1C10_11340	-	-	BT	-	-	-	-
	Hypothetical protein (Hsp90, PPR-repeats domains/protein aggregation, degradation & traffiking)	-	-	2	2	-	**CM**	MMMIC1C10_11350	-	BT	BT	BT	BT	BT	BT
	Hypothetical protein (ArsR-like domain, Winged helix-like DNA-binding/Transcriptional regulator)	A (arCOG01057**)**	-	-	-	-	C	MMMIC1C10_11370	1	BT	BT	BT	BT	BT	BT
	Hypothetical protein (MAM & Astacin-like domains)	-	-	-	-	-	C	MMMIC1C10_11380	1	-	-	-	-	-	-
	Hypothetical protein	-	-	-	-	-	C	MMMIC1C10_11390	1	-	-	-	-	-	-
	Hypothetical protein (Rhodanase-repeat domain/sulfur-transferase)	A (arCOG09529)	1,1	-	-	-	C	MMMIC1C10_11450	1	-	-	-	BT	-	BT
IV	Hypothetical protein (HxlR-like domain, Winged helix-like DNA-binding/Transcriptional regulator)	R (COG1733)	1,2	-	-	-	C	MMMIC1C10_11480	-	BT	BT	BT	BT	BT	BT
IV	Hypothetical protein	R (COG4325)	-	6	6	-	**CM**	MMMIC1C10_11490	2	BT	BT	BT	BT	BT	BT
IV	DEAD/DEAH box helicase family protein (DNA binding, endonuclease activity)	R (COG0610)	1,4	-	-	-	C	MMMIC1C10_11500	7	BT	BT	BT	BT	-	BT
IV	Type I restriction enzyme S subunit (DNA binding)	R (COG3465)	-	-	-	-	C	MMMIC1C10_11510	1	BT	BT	BT	BT	-	BT
IV	DNA Adenine N(4)/N(6)-Methyltransferase (Defense mechanims)	R (COG0286)	1,4	-	-	-	C	MMMIC1C10_11520	3	BT	BT	BT	BT	BT	BT
VI	Gfo/Idh/MocA Oxidoreductase (nucleotide binding)	R (COG0673**)**	1,2	-	-	-	C	MMMIC1C10_14420	-	-	-	-	BT	BT	-
VI	Hypothetical protein	B (ENOG502Z9SC)	1,0	-	-	-	C	MMMIC1C10_14430	2	-	-	-	-	-	-
VI	Archaeal glycosyltransferase family 4	R (COG0438)	1,5	-	-	-	**CM**	MMMIC1C10_14440	2	BT	BT	BT	BT	BT	BT
VI	Archaeal glycosyltransferase family 1	R (COG0438)	1,3	-	-	-	C	MMMIC1C10_14450	3	BT	BT	BT	BT	BT	BT
VI	Bacterial glycosyltransferase family 1	R (COG0438)	1,3	-	-	-	C	MMMIC1C10_14460	-	BT	BT	BT	BT	BT	BT
VI	DUF2206 domain-containing protein (transmembrane transport)	R (COG4906)	-	16	17	-	**CM**	MMMIC1C10_14470	6	-	-	-	BT	BT	-
	Hypothetical protein	-	-	-	-	-	C	MMMIC1C10_16090	1	-	-	-	-	-	-
VIII	Bacteriophage DNA packaging protein C (viral DNA genome packaging)	B (ENOG503174S)	0,4	-	-	-	C	MMMIC1C10_18330	-	-	-	-	-	-	-
VIII	Bacteriophage scaffold protein D (viral procapsid maturation)	B (ENOG502Z81Q)	0,9	-	-	-	C	MMMIC1C10_18340	-	-	-	-	-	-	-
VIII	Bacteriophage (Microviridae) spike protein F (viral capsid protein)	B (ENOG502Z8NM)	0,4	-	-	-	**E**	MMMIC1C10_18350	2	-	-	-	-	-	-
VIII	Bacteriophage major spike protein G (modulation by virus of host process)	B (ENOG502Z7SJ)	0,4	-	-	TAT	**E**	MMMIC1C10_18360	1	-	-	-	-	-	-
VIII	Bacteriophage minor spike protein H (symbiont entry into host cell)	B (ENOG502Z8AR)	0,4	-	-	SP	**E**	MMMIC1C10_18370	3	-	-	-	-	-	-
	Hypothetical protein (TPR_2 repeat, integral component of the membrane, ek.)	-	1,5	-	-	-	**E**	MMMIC1C10_18380	1	-	-	-	-	-	-
	Hypothetical protein (ek.)	-	-	-	-	-	**E**	MMMIC1C10_18390	-	-	-	-	-	-	-
	Hypothetical protein (ek.)	-	-	-	-	(SP)	**E**	MMMIC1C10_18410	1	-	-	-	-	-	-
	Hypothetical protein	-	-	-	-	-	C	MMMIC1C10_18440	-	-	-	-	-	-	-

Supplementary Table 6. Predicted N-linked glycosylation sites (NxS/T motifs) for cell-surface proteins of corrosive and non-corrosive *M. maripaludis* strains.

Threshold of protein identity >60% aminoacid sequence identity (aa. ID %) and >80% subject coverage. BT indicates values below threshold, dash indicates absence. 1.0. Non-corrosive strains (JJ, S2, C5) show higher divergence in glycosylation patterns for S-layer proteins. Signal peptide (SignalP 5.0) abbreviations: SP – standard signal peptides, LIPO – lipoprotein signal peptides, and TAT – TAT signal peptides. Cellular localization from DeepLocPro 1.0 abbreviations: C – cytoplasmic, CW – cell wall, CM – cell membrane, E – extracellular.

Number of N-linked glycosylation sites (N-X-S/T) by NetNGlyc 1.0. Glycosylation patterns of each strain's surface proteins were compared to those of Mic1c10, and variations are indicated in black boxes.

Annotation	Evolutionary rate	Signal peptide/LIPO/TAT	Localization	Mic1c10	NxS/T	OS7	% AA ID	NxS/T	KA1	% AA ID	NxS/T	MIC098Bin5	% AA ID	NxS/T	JJ	% AA ID	NxS/T	S2	% AA ID	NxS/T	C5	% AA ID	NxS/T
S-layer proteins (Sla)	1,5	SP	**E**	MMMIC1C10_09120	2	MMOS7_09490	100	2	MMKA1_09700	100	2	H0S71_00905	100	2	MMJJ_01180	93	3	MMP0875	BT	1	MmarC5_0683	BT	-
	1,5	SP	**E**	MMMIC1C10_12420	1	MMOS7_06120	99	1	MMKA1_06170	99	1	H0S71_07860	99	1	MMJJ_04150	95	-	MMP0569	98	-	MmarC5_1038	79	-
	1,5	SP	**E**	MMMIC1C10_14270	3	MMOS7_03960	62	3	MMKA1_04020	69	3	H0S71_06800	70	3	MMJJ_06560	70	2	MMP0383	61	-	MmarC5_1255	BT	-
S-layer family proteins(COG1361 - predicted component of the type IV pili system)	1,5	SP	**CM**	MMMIC1C10_07040	4	MMOS7_11150	96	4	MMKA1_11810	98	4	H0S71_01735	97	4	MMJJ_18330	97	4	MMP1021	97	4	MmarC5_0573	88	4
	1,5	SP	**CM**	MMMIC1C10_11730	3	MMOS7_06820	99	3	MMKA1_06870	99	3	H0S71_08210	99	3	MMJJ_03440	99	3	MMP0636	99	3	MmarC5_0968	92	3
	1,5	SP	**CM**	MMMIC1C10_12930	2	MMOS7_05620	98	2	MMKA1_05670	98	2	H0S71_07610	98	2	MMJJ_02120	BT	-	MMP0522	97	2	MmarC5_1085	BT	-
Cell-wall binding repeat containing protein	1,4	SP	**E**	MMMIC1C10_10740	2	MMOS7_07390	99	2	MMKA1_07460	99	3	H0S71_08500	99	3	MMJJ_02840	95	3	MMP0693	98	3	MmarC5_0884	BT	-
	1,4	SP	**CW**	MMMIC1C10_10750	16	MMOS7_07380	93	16	MMKA1_07450	100	16	H0S71_08495	100	16	MMJJ_02850	97	17	MMP0692	98	16	MmarC5_0884	89	14

Supplementary Table 7. List of annotated glycosyltransferases in the genomes of corrosive and non-corrosive *M. maripaludis* strains.

Threshold of protein identity >60% aminoacid sequence identity (aa. ID %) and >80% subject coverage. BT indicates values below threshold, dash indicates absence. Membrane helices from Interpro. Signal peptide (SignalP 5.0) abbreviations: SP – standard signal peptides, LIPO – lipoprotein signal peptides, and TAT – TAT signal peptides. Cellular localization from DeepLocPro 1.0 abbreviations: C – cytoplasmic, CW – cell wall, CM – cell membrane, E – extracellular. Number of N-linked glycosylation sites (N-X-S/T) by NetNGlyc 1.0.

Number of N-linked glycosylation sites (N-X-S/T) by NetNGlyc 1.0. Glycosylation patterns of each strain's glycosyltransferases were compared to those of Mic1c10, and variations are indicated in black boxes.

Annotated in:	Annotation (Primary functional categories)	PFAM	Evolutionary rate	TMHMM	PHOBIUS	Signal peptide/LIPO/TAT	Localization	Mic1c10		NxS/T	OS7		NxS/T	KA1		NxS/T	MIC098Bin5		NxS/T	JJ		NxS/T	S2		NxS/T	C5		NxS/T
Mic1c10	Archaeal oligosaccharyl transferase AglB (Cell wall/Membrane/Envelope biogenesis)	STT3 C & N terminal	1,5	13	14	SP	**CM**	MMMIC1C10_03010	100	3	MMOS7_15310	98	3	MMKA1_16480	98	3	H0S71_03820	97	3	MMJJ_14150	96	3	MMP1424	97	3	MmarC5_0154	77	4
	Glycosyltransferase, GT4 (Cell wall/Membrane/Envelope biogenesis/Carbohydrate Metabolism)	Glyco_trans_4	1,2	7	8	-	**CM**	MMMIC1C10_03030	100	1	MMOS7_15300	97	1	MMKA1_16470	97	1	H0S71_03815	96	1	MMJJ_14160	95	1	MMP1423	95	1	MmarC5_0155	86	1
	Glycosyltransferase, GT4 (Cell wall/Membrane/Envelope biogenesis/Carbohydrate Metabolism)	Glyco_trans_1 & 4	1,3	-	-	-	C	MMMIC1C10_04310	100	-	MMOS7_14000	100	-	MMKA1_15180	100	-	H0S71_03165	100	-	MMJJ_15500	99	-	MMP1293	98	-	MmarC5_0300	93	-
	Glycosyltransferase, GT2 (Signal Transduction/Motility/Carbohydrate Metabolism)	Glyco_trans_2	1,1	-	-	-	**CM**	MMMIC1C10_05520	100	1	MMOS7_12780	94	-	MMKA1_13480	98	-	H0S71_02540	99	1	MMJJ_16750	97	1	MMP1170	99	1	MmarC5_0417	94	-
	Glycosyltransferase, GT2 (Signal Transduction/Motility/Carbohydrate Metabolism)	Glyco_trans_2	-	1	1	-	**CM**	MMMIC1C10_06380	100	2	MMOS7_11950	100	2	BT	-	-	H0S71_02140	100	2	-	-	-	BT	-	-	BT	-	-
	Glycosyltransferase, GT4 (Cell wall/Membrane/Envelope biogenesis/Carbohydrate Metabolism)	Glyco_trans_1 & 4	1,5	-	-	-	**CM**	MMMIC1C10_06400	100	3	MMOS7_11930	100	3	BT	-	-	H0S71_02130	100	3	MMJJ_17630	83	3	BT	-	-	BT	-	-
	Glycosyltransferase, GT4 (Cell wall/Membrane/Envelope biogenesis/Carbohydrate Metabolism)	Glyco_trans_1 & 4	1,5	-	-	-	**CM**	MMMIC1C10_06420	100	5	MMOS7_11910	99	5	MMKA1_12590	85	4	H0S71_02120	100	5	BT	-	-	BT	-	-	BT	-	-
	Glycosyltransferase, GT2 (Signal Transduction/Motility/Carbohydrate Metabolism)	Glyco_trans_2, DPM/DPG-synthase	1,1	1	1	-	**CM**	MMMIC1C10_12210	100	-	MMOS7_06340	98	-	MMKA1_06390	100	-	H0S71_07965	100	-	MMJJ_03940	100	-	MMP0590	98	-	MmarC5_1016	94	-
	Glycosyltransferase, GT28 (Cell wall/membrane/envelope biogenesis/Peptidoglycan Synthesis)	Glyco_tran_28_C	1,3	-	-	-	**CM**	MMMIC1C10_14070	100	2	MMOS7_04180	99	4	MMKA1_04220	99	2	H0S71_06910	99	2	MMJJ_06340	99	2	MMP0403	99	2	MmarC5_1235	93	2
	Glycosyltransferase, GT2 (Signal Transduction/Motility/Carbohydrate Metabolism)	Glyco_trans_2, DPM/DPG-synthase	1,1	-	-	-	**CM**	MMMIC1C10_14400	100	2	BT	-	-	BT	-	-	H0S71_06735	99	2	BT	-	-	BT	-	-	MmarC5_1305	69	-
	Glycosyltransferase, GT4 (Cell wall/Membrane/Envelope biogenesis/Carbohydrate Metabolism)	Glyco_trans_1 & 4	1,5	-	-	-	**CM**	MMMIC1C10_14440	100	2	BT	-	-	BT	-	-	BT	-	-	BT	-	-	BT	-	-	BT	-	-
	Glycosyltransferase, GT1 (Cell wall/Membrane/Envelope biogenesis/Carbohydrate Metabolism)	Glyco_trans_1 & 4	1,3	-	-	-	C	MMMIC1C10_14450	100	3	BT	-	-	BT	-	-	BT	-	-	BT	-	-	BT	-	-	BT	-	-
	Glycosyltransferase, GT1 (Cell wall/Membrane/Envelope biogenesis/Carbohydrate Metabolism)	Glyco_trans_1 & 4	1,3	-	-	-	C	MMMIC1C10_14460	100	-	BT	-	-	BT	-	-	BT	-	-	BT	-	-	BT	-	-	BT	-	-
	Glycosyltransferase, GT2 (Cell wall/Membrane/Envelope biogenesis/Signal transduction)	Glyco_trans_2	1,3	-	-	-	**CM**	MMMIC1C10_14480	100	3	MMOS7_11950	67	2	BT	-	-	H0S71_02140	67	2	-	-	-	BT	-	-	MmarC5_1310	65	2
	Glycosyltransferase, GT4 (Cell wall/Membrane/Envelope biogenesis/Carbohydrate Metabolism)	Glyco_trans_1 & 4	1,3	-	-	-	C	MMMIC1C10_17270	100	3	MMOS7_01200	99	3	MMKA1_00950	99	3	H0S71_09020	99	3	MMJJ_10040	92	4	MMP0090	96	3	MmarC5_1586	86	5
OS7	Glycosyltransferase, GT4 (Cell wall/Membrane/Envelope biogenesis/Carbohydrate Metabolism)	Glyco_trans_1_4, GT4-PimA	-	-	-	-	C	MMMIC1C10_06410	100	1	MMOS7_11920	100	1	BT	-	-	H0S71_02125	100	1	BT	-	-	BT	-	-	BT	-	-
	Glycosyltransferase, GT5 (Glycogen-synthase/Cell wall/Membrane/Envelope biogenesis)	Glyco_trans_1 & 5	1,3	-	-	-	C	MMMIC1C10_04300	99	1	MMOS7_14010	100	1	MMKA1_15190	100	1	H0S71_03170	100	1	MMJJ_15490	99	1	MMP1294	99	1	MmarC5_0299	90	4
KA1	Glycosyltransferase, GT2 (Signal Transduction/Motility/Carbohydrate Metabolism)	Glyco_trans_2	1,3	1	1	-	**CM**	BT	-	-	BT	-	-	MMKA1_03830	100	1	BT	-	-	-	-	-	-	-	-	BT	-	-
	Glycosyltransferase, GT9 (Biosynthesis of lipooligosaccharides; Cell wall/Membrane/Envelope biogenesis)	GT9_LPS_heptosyltransferase	-	1	-	-	C	-	-	-	-	-	-	MMKA1_03840	100	1	-	-	-	BT	-	-	-	-	-	-	-	-
	Glycosyltransferase, GT4 (Cell wall/Membrane/Envelope biogenesis/Carbohydrate Metabolism)	Glyco_trans_1 & 4	1,5	-	-	-	**CM**	BT	-	-	BT	-	-	MMKA1_12600	100	-	BT	-	-	BT	-	-	BT	-	-	BT	-	-
	Glycosyltransferase, GT2 (Signal Transduction/Motility/Carbohydrate Metabolism)	Glyco_trans_2	1,3	-	-	-	**CM**	BT	-	-	BT	-	-	MMKA1_12610	100	1	BT	-	-	-	-	-	BT	-	-	BT	-	-
MAG	Glycosyltransferase, GT2 (Signal Transduction/Motility/Carbohydrate Metabolism)	Glyco_trans_2	1,1	1	1	-	**CM**	BT	-	-	BT	-	-	BT	-	-	H0S71_00135	100	-	BT	-	-	BT	-	-	MmarC5_1627	89	-
	Glycosyltransferase, GT1 (Cell wall/Membrane/Envelope biogenesis/Carbohydrate Metabolism)	Glyco_trans_1_4	1,3	-	-	-	**CM**	BT	-	-	BT	-	-	BT	-	-	H0S71_06725	100	3	BT	-	-	BT	-	-	MmarC5_1307	64	1
JJ	Glycosyltransferase, GT4 (Cell wall/Membrane/Envelope biogenesis/Carbohydrate Metabolism)	Glyco_trans_1_4, GT4-PimA	-	-	-	-	**CM**	BT	-	-	BT	-	-	BT	-	-	BT	-	-	MMJJ_17580	100	1	BT	-	-	BT	-	-
	Glycosyltransferase, GT9 (Biosynthesis of lipooligosaccharides; Cell wall/Membrane/Envelope biogenesis)	Glyco_trans_9	1,4	-	-	-	C	-	-	-	-	-	-	BT	-	-	-	-	-	MMJJ_17650	100	3	-	-	-	-	-	-
	Glycosyltransferase, GT9 (Biosynthesis of lipooligosaccharides; Cell wall/Membrane/Envelope biogenesis)	Glyco_trans_9	1,0	-	-	-	C	-	-	-	-	-	-	BT	-	-	-	-	-	MMJJ_17660	100	3	-	-	-	-	-	-
S2	Glycosyltransferase, GT1 (Cell wall/Membrane/Envelope biogenesis/Carbohydrate Metabolism)	Glyco_trans_1 & 4	1,5	-	-	-	**CM**	BT	-	-	BT	-	-	BT	-	-	BT	-	-	BT	-	-	MMP0356	100	3	BT	-	-
	Glycosyltransferase, GT2 (Signal Transduction/Motility/Carbohydrate Metabolism)	Glyco_trans_2	1,1	-	-	-	**CM**	BT	-	-	BT	-	-	BT	-	-	BT	-	-	BT	-	-	MMP0359	100	4	BT	-	-
	Glycosyltransferase, GT1 (Cell wall/Membrane/Envelope biogenesis/Carbohydrate Metabolism)	GT4-WbuB-like, Glyco-trans_4	1,3	-	-	-	**CM**	BT	-	-	BT	-	-	BT	-	-	BT	-	-	BT	-	-	MMP1080	100	-	BT	-	-
	Glycosyltransferase, GT1 (Cell wall/Membrane/Envelope biogenesis/Carbohydrate Metabolism)	Glyco_trans_1 & 4	1,5	-	-	-	C	BT	-	-	BT	-	-	BT	-	-	BT	-	-	BT	-	-	MMP1088	100	4	BT	-	-
C5	Glycosyltransferase, GT4 (Cell wall/Membrane/Envelope biogenesis/Carbohydrate Metabolism)	Glyco_trans_1_4, GT4-PimA	1,3	1	1	-	**CM**	BT	-	-	BT	-	-	BT	-	-	BT	-	-	BT	-	-	BT	-	-	MmarC5_0498	100	2
	Glycosyltransferase, GT1 (Cell wall/Membrane/Envelope biogenesis/Carbohydrate Metabolism)	Glyco_trans_1 & 1_4	1,3	-	-	-	C	BT	-	-	BT	-	-	BT	-	-	BT	-	-	BT	-	-	BT	-	-	MmarC5_1308	100	3
	**Total number of Glycosyltransferases/per genome**							**17**			**13**			**13**			**16**			**12**			**12**			**14**		
